# Identifying Baicalein as a Key Bioactive Compound in XueBiJing Targeting KEAP1: Implications for Antioxidant Effects

**DOI:** 10.3390/antiox14030248

**Published:** 2025-02-20

**Authors:** Ting-Syuan Lin, Xiao-Xuan Cai, Yi-Bing Wang, Jia-Tong Xu, Ji-Han Xiao, Hsi-Yuan Huang, Shang-Fu Li, Kun-Meng Liu, Ji-Hang Chen, Li-Ping Li, Jie Ni, Yi-Gang Chen, Zi-Hao Zhu, Jing Li, Yuan-Jia Hu, Hsien-Da Huang, Hua-Li Zuo, Yang-Chi-Dung Lin

**Affiliations:** 1 School of Medicine, The Chinese University of Hong Kong, Shenzhen, Shenzhen 518172, China; 222050501@link.cuhk.edu.cn (T.-S.L.); xiaoxuancai@link.cuhk.edu.cn (X.-X.C.); 222051029@link.cuhk.edu.cn (Y.-B.W.); jiatongxu@link.cuhk.edu.cn (J.-T.X.); 121090634@link.cuhk.edu.cn (J.-H.X.); huanghsiyuan@cuhk.edu.cn (H.-Y.H.); lishangfu@cuhk.edu.cn (S.-F.L.); chenjihang@cuhk.edu.cn (J.-H.C.); liliping@cuhk.edu.cn (L.-P.L.); jennyni@cuhk.edu.cn (J.N.); yigangchen@link.cuhk.edu.cn (Y.-G.C.); zihaozhu1@link.cuhk.edu.cn (Z.-H.Z.); lijing0903@cuhk.edu.cn (J.L.); huanghsienda@cuhk.edu.cn (H.-D.H.); 2 Warshel Institute for Computational Biology, School of Medicine, The Chinese University of Hong Kong, Shenzhen, Shenzhen 518172, China; 3 Guangdong Provincial Key Laboratory of Digital Biology and Drug Development, The Chinese University of Hong Kong, Shenzhen, Shenzhen 518172, China; 4 Center for Medical Artificial Intelligence, Shandong University of Traditional Chinese Medicine, Qingdao 266112, China; liukunmeng@sdutcm.edu.cn; 5 State Key Laboratory of Quality Research in Chinese Medicine, Institute of Chinese Medical Sciences, University of Macau, Macao 999078, China; yuanjiahu@um.edu.mo; 6 Department of Public Health and Medicinal Administration, Faculty of Health Sciences, University of Macau, Macao 999078, China; 7 Department of Endocrinology, Key Laboratory of Endocrinology of National Health Commission, Peking Union Medical College Hospital, Chinese Academy of Medical Sciences & Peking Union Medical College, Beijing 100730, China

**Keywords:** XueBiJing injection, NRF2/KEAP1 pathway, baicalein, oxidative stress, target identification, network pharmacology

## Abstract

Background: XueBiJing injection (XBJ) is renowned for its multi-target pharmacological effects, including immunomodulatory, antithrombotic, and antioxidant activities, offering potential therapeutic benefits for patients with severe infections such as sepsis and Coronavirus disease 2019 (COVID-19). Despite its clinical effectiveness, the molecular targets and mechanisms of XBJ remain unclear, warranting further investigation. Purpose: This study aimed to identify the key bioactive compounds in XBJ and elucidate their molecular targets and mechanisms. Methods: The zebrafish model was first used to evaluate the anti-inflammatory and antioxidant effects of XBJ, and the differentially expressed genes (DEGs) were identified by RNA sequencing and network analysis. Network pharmacology was used to analyze the relationship between bioactive compounds and molecular targets, and molecular docking and kinetic simulation were used to explore the target binding ability of key compounds. Cellular Thermal Shift Assay-Western Blot (CETSA-WB) and Surface Plasmon Resonance (SPR) further verified the interaction between compounds and targets; finally, the key pathways were confirmed by gene silencing experiments. Results: The zebrafish model results reveal that XBJ significantly reduced neutrophil and macrophage counts in a dose-dependent manner, emphasizing its potent anti-inflammatory effects. A transcriptomic analysis highlighted the differential expression of key genes in the KEAP1/NRF2 pathway, including *HMOX1*, *SLC7A11*, *NQO1*, and *TXNRD1*. A network analysis further pinpointed KEAP1 as a central molecular target, with tanshinone IIA, baicalein, and luteolin identified as key active compounds modulating this pathway. Among these, tanshinone IIA and baicalein exhibited strong binding interactions with KEAP1, which were confirmed through molecular docking and kinetic simulations. Further validation showed that baicalein directly targets KEAP1, as demonstrated by CETSA-WB and SPR analysis. Additionally, the gene silencing experiments of *KEAP1* and *NRF2* reinforced their crucial roles in activating the KEAP1/NRF2 pathway. Conclusion: These findings collectively establish baicalein as a critical bioactive compound in XBJ, driving its antioxidant and anti-inflammatory effects via KEAP1/NRF2 pathway activation through direct binding to KEAP1, providing new insights into the mechanism of action of XBJ.

## 1. Introduction

XueBiJing injection (XBJ) is an intravenous formulation derived from Chinese herbs and was approved by the National Medical Products Administration (NMPA) in 2004. It is used therapeutically to mitigate inflammation, modulate immune responses, and correct coagulopathies [1,2]. XBJ is formulated using extracts from *Carthamus tinctorius* L. (Carthami Flos, *hong hua*), *Paeonia lactiflora* Pall. (Paeoniae Radix Rubra, *chi shao*), *Ligusticum chuanxiong* Hort. (Chuanxiong Rhizoma, *chuan xiong*), *Salvia miltiorrhiza* Bge. (Salviae Miltiorrhizae Radix et Rhizoma, *dan shen*), and *Angelica sinensis* (Oliv.) Diels (Angelicae Sinensis Radix, *dang gui*) [3].

Among the bioactive components of XBJ, flavonoids play a particularly prominent role [4]. These flavonoids are well known for their capacity to modulate oxidative stress, inflammation, and immune responses, contributing to the multi-target, multi-pathway pharmacological effects of XBJ. Network pharmacology and molecular docking studies have identified many active compounds in XBJ, including key flavonoids such as baicalein and luteolin, along with other bioactive components such as tanshinone IIA, oxypaeoniflorin, and senkyunolide I. Collectively, these compounds enhance the therapeutic potential of XBJ [5,6]. Baicalein, derived from *Scutellaria baicalensis*, is a flavonoid with broad-spectrum antiviral and anti-inflammatory properties. It has demonstrated efficacy against various viruses, including influenza A (H1N1), Zika virus (ZIKV), and dengue virus (DENV). Baicalein has also been shown to reduce lung inflammation, inhibit pro-inflammatory cytokines such as IL-1β and TNF-α, and suppress reactive oxygen species (ROS) induced by SARS-CoV-2 [7,8,9]. These properties make baicalein a key flavonoid responsible for many of XBJ’s therapeutic effects.

XBJ exerts anti-inflammatory effects through several mechanisms, primarily by reducing oxidative damage in the lungs via the regulation of excess ROS [10]. Elevated ROS levels can directly damage proteins, lipids, and nucleic acids, leading to cell death [11,12,13,14]. ROS concentrations are controlled by antioxidant enzymes such as NAD(P)H quinone oxidoreductase-1 (NQO1) and heme oxygenase (HO), which are regulated by nuclear transcription factor NRF2 [15]. The KEAP1/NRF2 pathway is a therapeutic target for various diseases, particularly those associated with oxidative stress and inflammation. In certain diseases, the regulation of CERK and C1P has been shown to promote the dissociation of NRF2 from KEAP1 by competing with NRF2 for binding to the DGR domain of KEAP1. This process facilitates NRF2′s nuclear translocation [16]. Once inside the nucleus, NRF2 activates a series of target genes, including *HMOX1*, which encodes heme oxygenase-1 (HO-1). XBJ has been shown to upregulate both HO-1 expression at mRNA and protein levels [17]. HO-1 has been identified as a key hub gene in this pathway, playing a vital role in cellular defense against oxidative damage. Furthermore, evidence suggests that HO-1 can inhibit viral replication, further highlighting the therapeutic potential of modulating the KEAP1/NRF2 pathway [18,19,20]. Moreover, previous research on the chemical composition of XBJ revealed that quercetin, ferulic acid, kaempferol, and paeoniflorin can downregulate NFκB1 and NFκB2 expression, which are overexpressed in patients with sepsis and may drive the overproduction of inflammatory cytokines [21]. These findings suggest that XBJ contains multi-component, multi-target, and multi-pathway characteristics, which provide a precise molecular basis for modulating biologically active pathways, indicating that its effects are mediated through immune regulation and antioxidative stress mechanisms [2].

**Figure 1:**
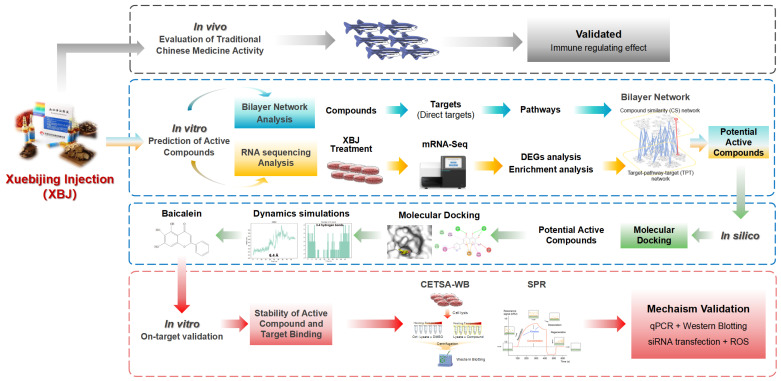
Workflow of investigation on XueBiJing (XBJ) injection.

However, the exact mechanisms through which these constituents modulate immune response and contribute to therapeutic effects within the context of this herbal medicine, including their direct targets, are not well understood. Herein, the primary goal of this research is to identify the key bioactive compounds in XBJ and their target, with a focus on understanding the molecular mechanisms underlying XBJ’s immunomodulatory and antioxidant effects. The findings have significant potential to impact the domains of TCM, immunology, and pharmaceutical development, thereby contributing to enhanced patient outcomes and the innovation of novel therapeutic strategies. The workflow of this study is shown below (Figure 1).

## 2. Materials and Methods

### 2.1. Materials

XBJ, a sterile and nonpyrogenic injection for intravenous administration, is manufactured by Tianjin Chase Sun Pharmaceutical Co., Ltd. (Tianjin, China), and was purchased from Shijiazhuang Kang Ren Tang Pharmaceutical Co., Ltd., Shijiazhuang, China (No.2107191). UPLC-QTOF-MS was conducted to identify the compounds in XBJ. The detailed parameters of the compounds are listed in the Supplementary Information presenting the LC-MS/MS analysis of XBJ [22]. Standard compounds, namely oxypaeoniflorin (No. DY0074-0020, molecular weight (MW): 496.46 g/mol), senkyunolide I (No. DY0009-0020, MW: 224.25 g/mol), luteolin (No. DM0032-0020, MW: 286.24 g/mol), tanshinone IIA (No. DD0011, MW: 294.34 g/mol), and baicalein (No. DH0024-0020, MW: 270.24 g/mol), with a purity of 98%, were purchased from Chengdu Desite Biological Technology Co., Ltd., China (Chengdu, China). The identification results of XBJ by UPLC-Q-TOF-MS/MS was listed in the Supplementary Table S3.

### 2.2. In Vivo Evaluation

To investigate the immune regulating effects of XBJ, we conducted an in vivo evaluation using a zebrafish model of a Poly (I:C)-induced infection. The International Association for Assessment and Accreditation of Laboratory Animal Care (AAALAC, 001458) accredited Hangzhou Hunter Biotechnology, Inc. (Hangzhou, China) for conducting the zebrafish experiments, and the company was granted permission to use experimental animals (SYXK [Z]2022-0004).

#### 2.2.1. Zebrafish Models

Zebrafish were maintained in water containing 0.02% instant ocean salt, with a pH of 6.5~8.5, an electrical conductivity of 450~550 μS/cm, and a hardness of 50~100 mg/L CaCO_3_, at 28 °C.

#### 2.2.2. Poly (I:C)-Induced Viral Infection Model (Neutrophils)

A transgenic zebrafish line for the in vivo visualization of neutrophil myeloperoxidase (MPX) was used, and embryos were obtained from natural crosses of mating pairs. A total of 5 dpf MPX transgenic zebrafish were used for further experiments. We randomly selected samples and placed them onto a 6-well plate, where each well (3 mL) contained 30 tails of zebrafish. The samples were administered in water (for concentration, see Supplementary Table S1), a normal control group and a model control group were set up simultaneously, and the volume of each well was 3 mL. A positive control of dexamethasone acetate at a concentration of 100 μM was also included. Except for the normal control group, all other experimental groups were given Poly (I:C) (100 ng/fish) by swim bladder injection to establish a zebrafish model of viral inflammation. After being treated for 3 h at 28 °C, 10 zebrafish from each group were randomly selected to be observed, and optical images were captured under a fluorescent microscope (Nikon AZ100, Minato City, Japan) and then analyzed by image processing software (NIS-Elements D 3.20). The number of neutrophils in swim bladder was used to evaluate the efficacy in resisting viral pneumonia (neutrophils). The statistical results were expressed by mean ± standard errors (SE), and *p* < 0.05 indicated that the difference was statistically significant.

#### 2.2.3. Poly (I:C)-Induced Viral Infection Model (Macrophage)

A transgenic zebrafish line for the in vivo visualization of macrophage myeloperoxidase (MPX) was used, and embryos were obtained from natural crosses of mating pairs. A total of 5 dpf MPX transgenic zebrafish were used for further experiments. We randomly selected samples and placed them onto a 6-well plate, with each well (3 mL) containing 30 tails of zebrafish. The samples were given in water (for concentration, see Supplementary Table S1) with a positive control of dexamethasone acetate at a concentration of 100 μM, a normal control group and a model control group were set up simultaneously, and the volume of each well was 3 mL. Except for the normal control group, all other experimental groups were given Poly (I:C) (100 ng/fish) by swim bladder injection to establish a zebrafish viral inflammation model. After being treated for 3 h at 28 °C, 10 zebrafish from each group were randomly selected to be observed, and optical images were captured under a fluorescent microscope (Nikon AZ100, Minato City, Japan) and then analyzed by image processing software (NIS-Elements D 3.20). The fluorescence intensity of macrophages in swim bladder were used to evaluate the efficacy in resisting viral pneumonia (macrophages). The statistical results were expressed as mean ± SE, and *p*-values < 0.05 indicated that the difference was statistically significant.

### 2.3. Cell Culture

MCF-7 cells (Cellcook Biotech Co., Ltd., Guangzhou, China) were regularly cultured in MEM media (Gibco, Billings, MT, USA, No. 31095029) supplemented with 10% FBS (Gibco, No. 26170043), 1×Non-Essential Amino Acid solution (Gibco, Billings, MT, USA, No. 11140050), and 10 μg/mL insulin (CAS: 11061-68-0, Nanjing Duly Biotech Co., Ltd., Nanjing, China) and incubated at 37 °C in a humidified environment with 5% CO_2_. Trypsin-EDTA was used to detach and subculture the cells.

### 2.4. CCK-8 Assay and Drug Treatment

To find the applicable range of XBJ to MCF-7 cells, CCK8 was used to detect the activity curve of MCF-7. XBJ and the potential active compounds were diluted or dissolved in MCF-7 culture medium (total 2.0 × 10^4^ cells/well) in 96-well plates. After 48 h of incubation, the suspension was replaced by fresh medium (Control group) or drug solution (treatment groups) at a series of doses. After another 24 h of incubation, the suspension was replaced by 100 μL/well CCK-8 (Beyotime, Shanghai, China No. C0038) solution at a ratio of 1:10 and incubated for 2 h at 37 °C. The absorbance was measured at a 450 nm wavelength using a microplate reader (BioTek Epoch 2, Santa Clara, CA, USA).

Prior to being exchanged with medium containing XBJ/compounds at three different concentrations (high-IC50, medium-IC30, and low-IC10) and a model control with two experimental repeats, MCF-7 cells were plated at a density of 3.34 × 10^5^/mL cells per 10 cm dish and incubated for 48 h. The next day, 1 mL of TRIzol reagent (Invitrogen, Carlsbad, CA, USA No. 15596026) per dish was used to collect the cells. MCF-7 cells’ total RNA was isolated using the RNA Isolation Kit (Direct-zol^TM^ RNA Miniprep Kits, Irvine, CA, USA No. R2052). Before undergoing additional processing, the extracted RNA was dissolved in RNase-free water and kept at −80 °C. A spectrophotometer (Implen NanoPhotometer^®^ N60/N50, Munich, Germany) was used to measure the concentration of RNA.

### 2.5. Transcriptomic and Enrichment Analysis

Transcriptomic analysis was performed to identify differentially expressed genes and potential pathways under different drug concentrations. RNA-seq was conducted using BGISEQ500 platform using 2 × 150 bp, paired end. The alignment and filtering of reads were conducted by nf-core/rna-seq v3.4 [23] with “--skip_qualimap” and other default parameters, reference genome FASTA for GRCh38, and annotation gtf file from genecode v39.

For differential expression and pathway analysis, iDEP v0.96 [24] was used. Genes with expressions smaller than 0.5 count per million (CPM) in all samples were excluded for further analysis. R package DESeq2 v1.46.0 in built iDEP was selected for differential expression analysis. When comparing samples treated with XBJ to non-treated ones, DEGs were found with |log_2_FC| ≥ 1 and *p* < 0.05. Enrichment analysis was carried out using MetaCore^TM^ (©2022 Clarivate Analytics, Philadelphia, PA, USA) with enriched threshold, *p* < 0.05.

### 2.6. Network Construction and Analysis

In order to identify the major compounds and their potential targets, a Compounds’ similarity (CS) - Target-protein-target (TPT) bilayer network analysis was employed. This is an updated method based on our previous work [25,26] and is included in the Supplementary Information in the network analysis.

### 2.7. Real-Time qPCR

The mRNA expression levels of identified DEGs, such as *HMOX1*, *CYP1A1*, *SLC7A11*, *NQO1*, and *TXNRD1*, were examined using quantitative PCR (qPCR) in samples of MCF-7 cells. The primer sequences used for qPCR are listed in Table 1. Total RNA was extracted using the Direct-zol^TM^ RNA miniprep kit (Irvine, CA, USA No. R2052) in accordance with the manufacturer’s instructions. Next, cDNA was generated using SuperScript III Reverse Transcriptase (Invitrogen, Carlsbad, CA, USA No. 18080093). The master mixture and SYBR Green were combined with total cDNA, which was then loaded into the Applied Biosystems QuantStudio^TM^ 6 Flex Real-Time PCR System (Carlsbad, CA, USA) for amplification and detection.

### 2.8. Western Blotting

To validate differential protein expression corresponding to genes, Western blotting was carried out as previously mentioned [27]. An equivalent quantity of protein was separated using 8% SDS-PAGE and subsequently put onto membranes made of polyvinylidene difluoride (Millipore, Burlington, MA, USA) using the Genscript eBlot L1 wet transfer system (GenScript, Nanjing, China No. L00686). After that, transferred membranes were blocked for 15 min at room temperature using EveryBlot Blocking Buffer (BIO-RAD, Hercules, CA, USA No. 12010947). Then, membranes were probed at 4 °C overnight with the following specific primary antibodies: anti-HO-1 (ABCAM, Cambridgeshire, UK No. ab68477), anti-NQO1 (ABCAM, Cambridgeshire, UK No. ab80588), anti-SLC7A11 (ABCAM, Cambridgeshire, UK No. ab307601), anti-NRF2 (ABCAM, Cambridgeshire, UK No. ab62352), anti-KEAP1 (ABCAM, Cambridgeshire, UK No. ab227828), and anti-GAPDH (Proteintech, Rosemont, IL, USA No. 60004-1-lg). After that, secondary antibodies conjugated to horseradish peroxidase were added to the membranes (Cell Signaling Technology, Danvers, MA, USA No. 7076P2/7074P2). After reacting with electrochemiluminescence (ECL, Immobilon Western, Millipore, Burlington, MA, USA), the chemiluminescence imaging was ultimately detected by the eBlot Touch Imagertm (eBlot Photoelectric Technology, Shanghai, China). The ImageJ software v1.49 (US National Institutes of Health, NIH, Bethesda, Rockville, MD, USA) was used to quantify densitometric estimations.

### 2.9. Molecular Docking, Dynamics Simulations, and Trajectory Data Analysis

Molecular docking was performed to evaluate the binding potential of predicted compounds with targets. Protein–ligand complex crystal structures with an X-ray resolution of less than 3 Å were sourced from the Protein Data Bank (RCSB PDB, www.rcsb.org/) [28]. The 3D coordinates of anticipated active compounds were retrieved from the PubChem database. The structures of proteins and corresponding compounds were then docked using Discovery studio in conjunction with AutoDockTools 1.5.6 (ADT), specifically AutoDock Vina, a molecular docking and virtual screening program [29]. The target protein’s active site was identified as the binding pocket of the native ligand (ML334, PubChem CID: 56840728, CAS No. 1432500-66-7) in the crystal structure [30]. As positive controls, the native ligand and the reported NRF2 activator (KEAP1 binder) [31] were used to guarantee the accuracy of the molecular docking active site. Furthermore, Discovery Studio 4.5 produced the 2D interaction diagrams that allowed researchers to visualize the calculated interactions between the residues of KEAP1 and the anticipated active compounds.

To understand the dynamic stability of anticipated active compounds binding to targets, molecular dynamics (MD) simulations were carried out in GROMACS 2023.3 software [32,33]. Topology files of small compounds of interest were generated using the CGenFF program [34,35]. The introduction of solvation effects on the investigated protein–ligand complexes was performed by implementing the TIP3P solvation model [36] using the Monte-Carlo Ion Placing Method, and the solvated system was neutralized with 0.15M Na^+^ and Cl^−^ ions. All simulations were carried out using the CHARMM36 force field [37]. The steepest decent energy minimization with 5000 steps with a tolerance of up to 1000 kJ/mol^−1^nm^−1^ was conducted for each system under NVT ensemble conditions. System equilibrium was carried out under an NVT ensemble using a Noose–Hoover thermostat to 310.15 K. The MD process was conducted in an NPT ensemble for a 50 ns time scale with the implementation of a *Parrinello–Rahman barostat* [38] (T_p_ = 5 ps) and a *Noose–Hoover thermostat* [39] (T_t_ = 1 ps). The LINCS algorithm [40] constrains the covalent bonding of hydrogen atoms.

The root mean square deviation (RMSD) value of the whole system and the distance between ligands and protein were calculated using the “rms” module of GROMACS. The number of hydrogen bonds between the compounds and proteins during MDS was calculated using the “hbond” module. Binding energies of the protein–ligand complexes were calculated by following the Molecular Mechanics Poisson–Boltzmann Surface Area (MM/PBSA) protocol using the GB model.

### 2.10. Cellular Thermal Shift Assay—Western Blotting (CETSA-WB)

CETSA-WB experiments were performed to verify the relationship between potential active compounds and KEAP1. MCF-7 cells were treated with RIPA lysis buffer (Beyotime, Shanghai, China No. P0013C) in order to extract soluble proteins. Equal amounts of proteins were then incubated with baicalein and tanshinone IIA as the treatment group, or with DMSO as the negative control group, at room temperature for 10 min prior to the CETSA heat pulse. The samples were then aliquoted into PCR tubes. The samples were subjected to heating at different temperatures ranging from 37 °C to 70 °C using an Applied Biosystems PCR analyzer (Thermo Scientific, Waltham, MA, USA). After heating for 3 min, the samples were cooled at 4 °C for 10 min. Subsequently, the heated samples were diluted with 160 μL of DNase-free water, mixed thoroughly, and transferred into centrifuged tubes.

Subsequently, the samples were subjected to centrifugation at 20,000 × *g* for 30 min at 4 °C. The resulting supernatant was mixed with 6 × loading buffer and then denatured at 100 °C for 10 min. The denatured samples were quickly transferred to ice and cooled for an additional 10 min. Following cooling, the samples were centrifuged for 10 min at 4 °C at 14,000 × *g*, and the supernatant was removed to be loaded onto a gel for Western blot analysis.

### 2.11. Surface Plasmon Resonance (SPR) Analysis

SPR experiments on the interaction of KEAP1 (Sino Biological Inc., Beijing, China, No. 11981-HNCB) with potential active compounds were performed using a Biacore X100 (Piscataway, NJ, USA) optical biosensor. Immobilization of the recombinant KEAP1 protein (57.6 μg/mL) was performed in sodium acetate buffer (pH 4.0). During each binding cycle, the analyte solution (0, 1.5625, 3.125, 6.25, 12.5, 25, 50, 100, and 200 μM) was injected at a flow rate of 20 μL/min for 1 min, and dissociation was monitored for 150 s. Data collection and organization were consistent with the standardized operation.

### 2.12. siRNA Transfection and ROS Measurement

We aimed to verify the silencing effect of *KEAP1* and *NRF2* and to explore their effect on downstream gene expression, and the siRNA oligo list is presented in Supplementary Information Table S2. We used Lipofectamine™ 2000 Reagent (Thermo Fisher, Waltham, MA, USA No. 11668027) to transfect siRNA targeting *NRF2* (siNRF2) and *KEAP1* (siKEAP1) to suppress the expression of *NRF2* and *KEAP1* genes in cells, with non-coding RNA (NCR) serving as a negative control. The siRNA oligonucleotides were synthesized by IBSBIO (Shanghai, China), and their sequences are provided in Table S2 and Figure S2. Cell transfection was conducted following the manufacturer’s instructions at a concentration of 50 nM siRNA. The Lipofectamine™ 2000 reagent and siRNA diluent were mixed for 20 min at room temperature. The cells were treated with siRNA for 48 h and then with a compound treatment for another 24 h. The transfected cells were divided into different groups, including control groups without treatment or baicalein group, NCR group, positive control group, siNRF2 groups with baicalein, siKEAP1 groups with baicalein, and untreated drug group. Following treatment, cells were collected for subsequent qPCR analysis and ROS measurement. ROS were measured by H2DCFDA at 37 °C for 25 min after siRNA and drug treatment. Then, the cells were collected and analyzed using a flow cytometer (CytoFLEX S, Beckman Coulter, Brea, CA, USA).

## 3. Results

### 3.1. Anti-Inflammatory Effect of XBJ on Poly (I:C)-Induced Infection

**Figure 2:**
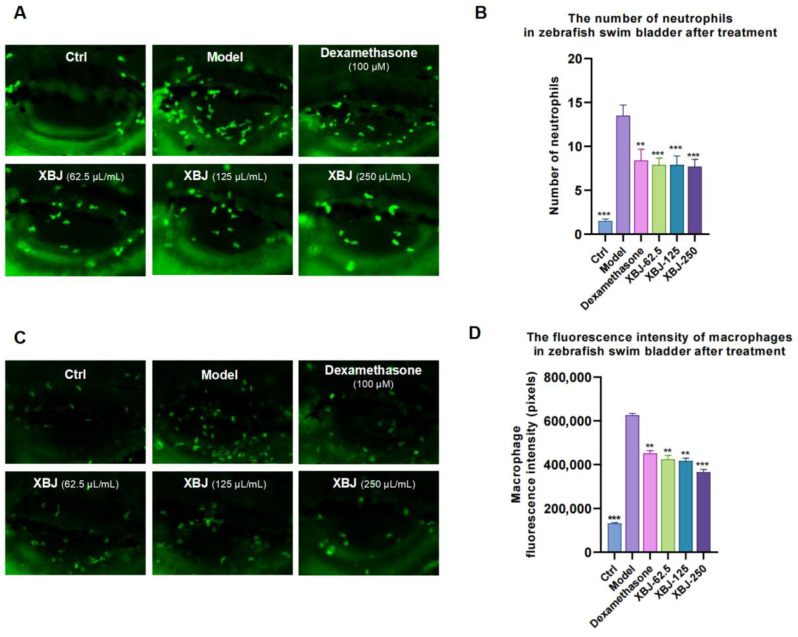
The effect of XBJ on neutrophils and macrophages. (**A**) A representative image showing fluorescence detection on neutrophils, captured using a fluorescent microscope (Nikon AZ100, Minato City, Japan). (**B**) The number of neutrophils in zebrafish swim bladder after treatment. (**C**) A representative image showing fluorescence detection on macrophages, captured using a fluorescent microscope (Nikon AZ100, Minato City, Japan). (**D**) The fluorescence intensity of macrophages in zebrafish swim bladder after treatment. In all experimental trials, a minimum of 30 larvae were utilized for each condition. Statistical significance is denoted by *** for *p* < 0.001 and ** for *p* < 0.01 when compared to the Dexamethasone group.

In the first section of this study, we evaluated the effects of XBJ on the innate immune response of a zebrafish model induced by Poly (I:C). Poly (I:C) is a synthetic double-stranded RNA that activates toll-like receptor 3 (TLR3), simulating viral infection [41]. This activation triggers an innate immune response, leading to inflammation and tissue damage. Neutrophils, the predominant leukocytes, are essential for the innate immune response and are the first-line defense against infections, engaging in pathogen phagocytosis at infection sites. Macrophages are equally crucial, phagocytosing pathogens and triggering inflammation via cytokine release, being vital for infection containment, tissue protection, and healing. Both cell types are instrumental in pathogen clearance and drive the inflammatory response for an effective immune reaction. In the swim bladder of zebrafish, poly (I:C) injection increased neutrophil and macrophage numbers and fluorescence intensity, indicating an inflammatory response. XBJ treatment significantly reduced the numbers of both neutrophils (Figure 2A,B) and the fluorescence intensity of macrophages (Figure 2C,D) in a dose-dependent manner, suggesting an anti-inflammatory effect. Additionally, XBJ showed superior efficacy compared with dexamethasone, a positive control drug which has anti-inflammatory properties.

### 3.2. Genes Involved in NRF2-Regulated Oxidative Stress Response Were Inferred by XBJ

**Figure 3:**
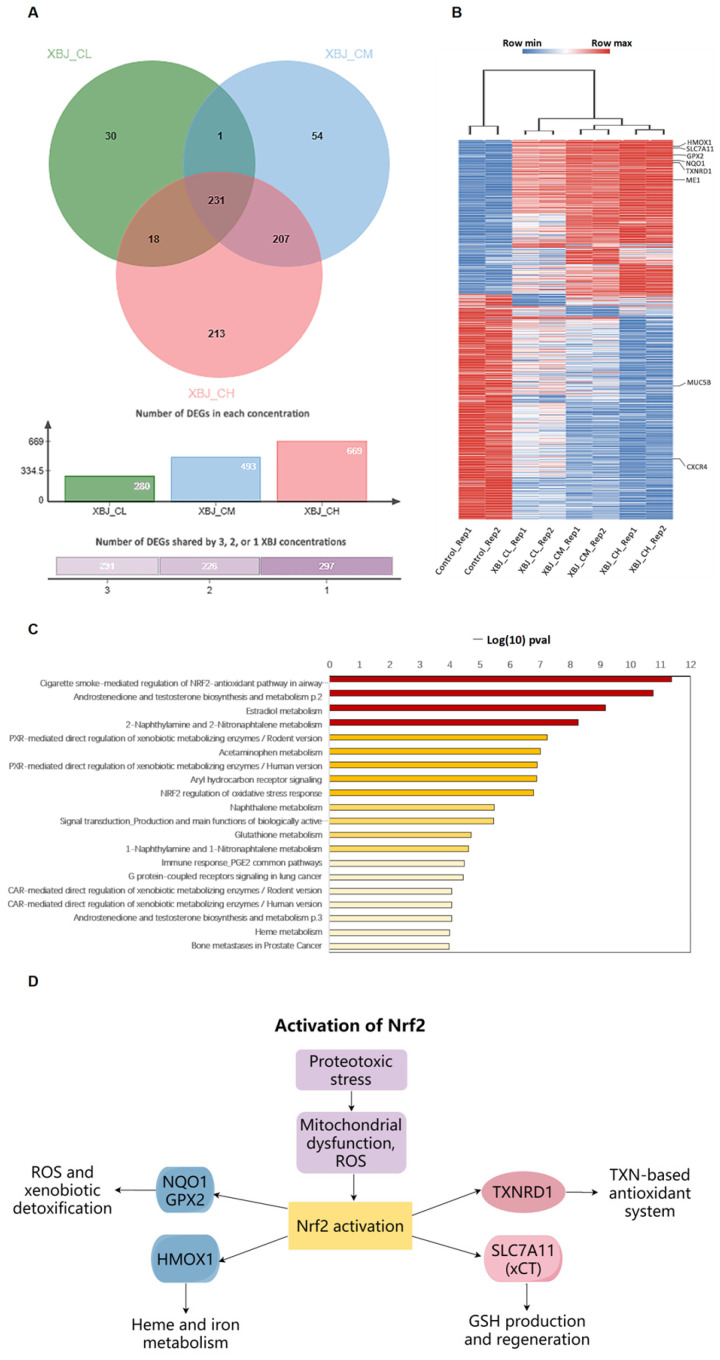
Results from RNA sequencing data analysis of samples treated with XBJ. (**A**) Differential expression analysis of low, medium, and high concentrations. (**B**) Heatmap visually represents expression patterns of differentially expressed genes. (**C**) Functional enrichment analysis indicates significant correlation between these differentially expressed genes and specific biological pathways. (**D**) Activation pathways of Nrf2 and its role in various cellular processes.

The differential expression analysis results indicate that the number of DEGs increases as the concentration of XBJ increases. Samples treated with XBJ in a high concentration (CH) have 669 DEGs, with 493 DEGs in a medium concentration (CM) and 280 DEGs in a low concentration (CL) compared to the non-treated samples. The number of DEGs intersecting the three concentrations was 231 (Figure 3A). The expression profiles of DEGs are shown as a heatmap, and an increasing concentration of XBJ shows stronger regulation in gene expressions (Figure 3B). Within these DEGs, several genes were reported to be associated with patients with severe infections. The expression of *MUC5B* and *CXCR4* decreases as the concentration of XBJ increases. *MUC5B* overexpression in lung tissue is linked to muco-ciliary dysfunction and fibrosis in mice [42,43,44]. Patients with severe infection show elevated *CXCR4* expression in neutrophils [45].

A functional enrichment analysis shows that these DEGs are highly related to coagulation processes, such as “signal transduction production and main functions of biologically active prostaglandins and Thromboxane A2” and “Inflammation associated coagulopathy” (Figure 3C). The inference of an immune response is another main function related with DEGs, such as the immune response involving *PGE2*, *IL-1*, *IL-4*, and complement regulation. Within the top 200 enriched pathways, 42 pathways are related to the inference of coagulation and/or immune response (Supplementary Table S4). These results indicate that XBJ mainly treats pneumonia by inferring pathways related to coagulation and immune response.

The NRF2 regulation of oxidative stress response is one of the pathways enriched by DEGs with top1 and top9 *p*-value ranking. The activation of NRF2 and its downstream pathways was related to antioxidant effects and the anti-inflammation response by inhibiting the secretion of pro-inflammatory cytokines [46,47]. The activation of NRF2 and its downstream pathway has been heatedly discussed as one of the potential therapeutic strategies for patients with severe infection [46,48]. *HMOX1*, *SLC7A11*, *NQO1*, and *TXNRD1* are genes that are involved in the NRF2 regulation of the oxidative stress response (Figure 3D).

### 3.3. Conducting a Network Analysis to Identify the Potential Active Ingredients

#### 3.3.1. CS Network Construction and Network Analysis

**Figure 4:**
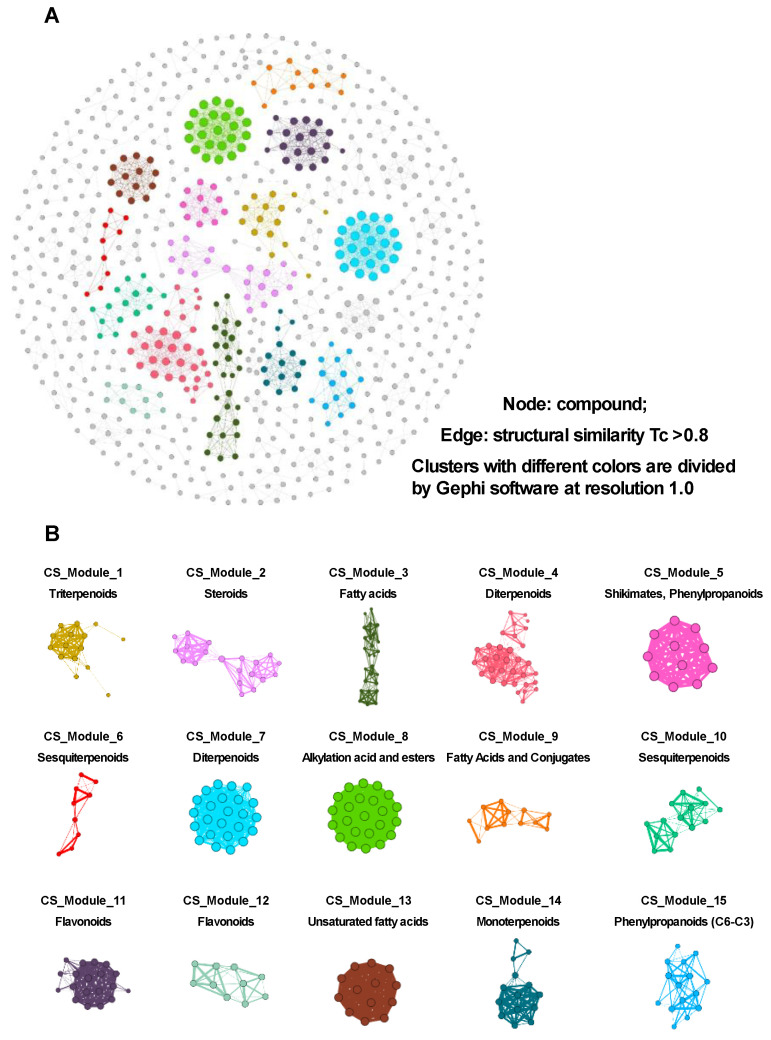
The CS network of XBJ. (**A**) CS network of XBJ was established based on 701 compounds. (**B**) Primary constituents of XBJ within each module exhibit substantial structural similarities.

A comprehensive collection of 1069 XBJ compounds was obtained from databases and relevant studies in the literature. A total of 701 of these compounds have structural similarity (*Tc* ≥ 0.8), and the SuperPred, SEA, and TCMSP analyses identified them with at least one predicted human target. Additionally, as illustrated in Figure 4A, the phytochemical compound profile, or the CS network of XBJ, was developed using these 701 compounds. The compounds are represented by nodes in a CS network, and associations between pairs of compounds with a *Tc* value of 0.8 or higher are represented by edges. This suggests that compounds with edges exhibit strong structural resemblances. Using Gephi’s 1.0 resolution implementation of the Louvain algorithm, the clusters within the CS network were located and split. A brief analysis of the results is displayed in Figure 4B. The structures of nodes (compounds) within each cluster were examined separately using the chemical data from PubChem. With 701 nodes and 1958 edges, the CS network of XBJ was segmented into multiple modules. The compounds in each module are very similar in structure, and the main constituents of XBJ are summarized in Figure 4B, including triterpenoids (CS_module_1), steroids (CS_module_2), fatty acids (CS_module_3), diterpenoids (CS_module_4), shikimates or phenylpropanoids (CS_module_5), sesquiterpenoids (CS_module_6), diterpenoids (CS_module_7), alkylation acid and esters (CS_module_8), fatty acids and conjugates (CS_module_9), sesquiterpenoids (CS_module_10), flavonoids (CS_module_11 and CS_module_12), unsaturated fatty acids (CS_module_13), monoterpenoids (CS_module_14), phenylpropanoids (C6-C3) (CS_module_15), and some compounds with various structures (CS_others). The size of a node is proportional to its degree.

#### 3.3.2. TPT Network Construction and Network Analysis

**Figure 5:**
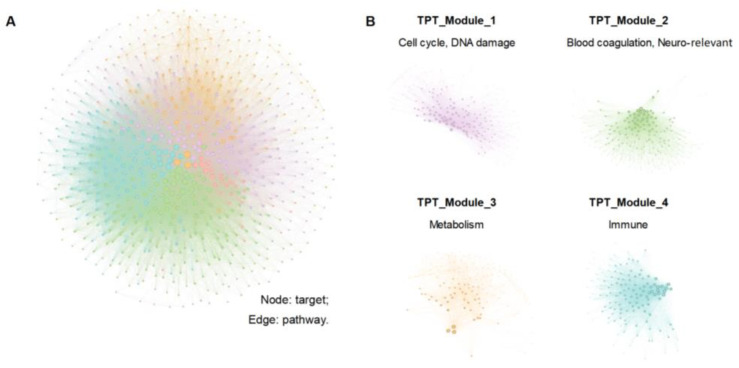
The TPT network of XBJ. (**A**) In total, 847 human targets are associated with 701 compounds, and 683 protein targets were enriched to pathways with *p* < 0.05. (**B**) The TPT network decomposed into four major modules.

As in our previous study, the TPT network was created to uncover the underlying functional property of enormous targets through a network module-based analysis [49,50,51]. SuperPred, SEA, and TCMSP predicted that 847 human targets would be associated with 701 compounds for the TPT network analysis of XBJ. Based on Metacore, 683 of these protein targets were enriched to pathways with *p* < 0.05. The target–pathway interactions were also utilized to create the TPT network, which Gephi’s Louvain algorithm then detected. As shown in Figure 5A, the TPT network decomposed into four major modules, as shown in Figure 5B, and their functional propensity, as evaluated using the previous method, indicate that the targets in TPT_Module_1 mainly participate in cell cycle or DNA damage, the targets in TPT_Module_2 mainly participate in blood coagulation, a neuro-relevant pathway, the targets in TPT_Module_3 are mainly involved in metabolism, and the targets in TPT_Module_4 are mainly immune-relevant. The size of a node is proportional to its degree (Supplementary Information Table S5).

#### 3.3.3. Bilayer Network Analysis and Selected Important Compounds

**Figure 6:**
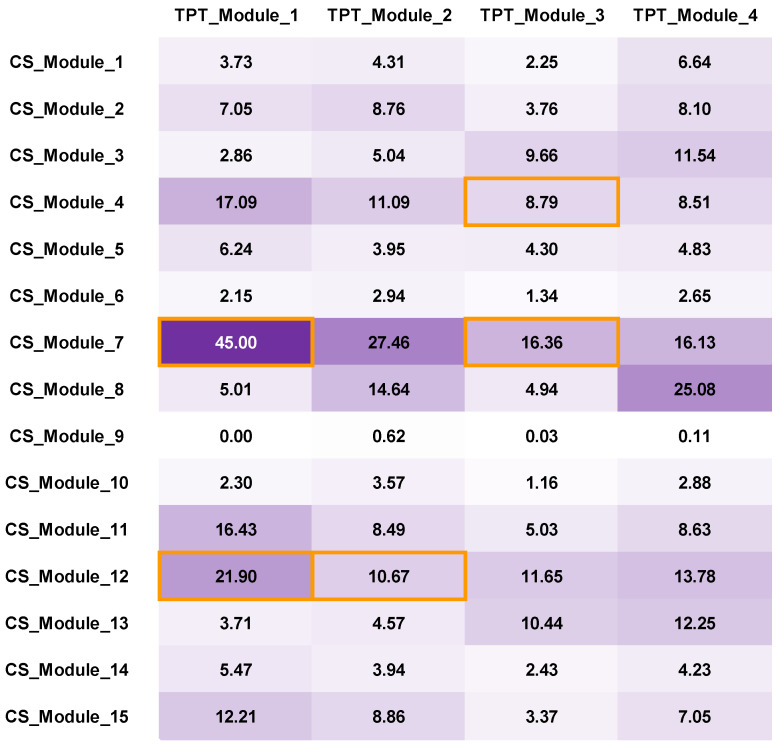
The calculated association strength (***e_uv_***) of each compound module with each target module. The purple and white refer to the highest and lowest ***e_uv_*** values, respectively. The values highlighted with an orange frame denote the locations of the targets that participated in the pathway (NRF2 regulation of oxidative stress response) we are interested in within the TPT network and the locations of the compounds that are associated with these targets in the CS network.

The bilayer CS-TPT network was established based on compound–target interactions. A subsequent network analysis was further conducted to identify the compounds with higher contribution by taking the network module and the weight of each node into consideration. Since multiple compounds may act with multiple targets, multi-compound modules may act with multi-target modules. As illustrated in Materials and Methods, we used ***e_uv_*** to measure the association between the compound module in the CS network (***m_u_***) and the target module in the TPT network ($m_{v}^{*}$). Similarly, ***e_uv_*** can be calculated by summing ***d_ij_***. The ***e_uv_*** value reflects the strength of the association between compound modules (CS_Module_1~CS_Module_15) and target modules (TPT_Module_1~ TPT_Module_4), as shown in Figure 6, where the purple and white refer to the highest and lowest ***e_uv_*** values, respectively. In detail, Figure 6 denotes that the diterpenoids in CS_Module_7 exhibited the most significant association with the targets in TPT_Module_1, while the flavonoids in CS_Module_12 demonstrated a higher likelihood of regulating the targets in TPT_Module_1, which are functionally relevant to the cell cycle or DNA damage. The diterpenoids in CS_Module_7 and CS_Module_4, alkylation acid and esters in CS_Module_8, and flavonoids in CS_Module_12 displayed a stronger association with TPT_Module_2, and they are functionally relevant to blood coagulation and neuro-relevant function. The diterpenoids in CS_Module_7, fatty acids in CS_Module_3, flavonoids in CS_Module_12, and unsaturated fatty acids in CS_Module_13 displayed a higher association with TPT_Module_3, and they are the most relevant to metabolism. TPT_Module_4, which is highly relevant to immunity, is mainly associated with the diterpenoids in CS_Module_7 and alkylation acid and esters in CS_Module_8.

For the NRF2 regulation of the oxidative stress response pathway that we are interested in, the targets involved in this pathway and the compounds that may interact with these targets are highlighted in the orange frame in Figure 6. Furthermore, for each pair of investigated compound module (***m_u_***) and target module ($m_{v}^{*}$), every single compound’s contribution ***C(i)_uv_*** to the target module was calculated, and the results are displayed in Table S2. Ranking according to the ***C(i)_uv_*** values, we selected the top three potential active compounds with the highest ***C(i)_uv_*** values for subsequent verification, including tanshinone IIA, luteolin, and baicalein.

### 3.4. Molecular Docking and Dynamic Simulation Insights into the Binding Efficacy of Predicted Compounds with KEAP1

**Figure 7:**
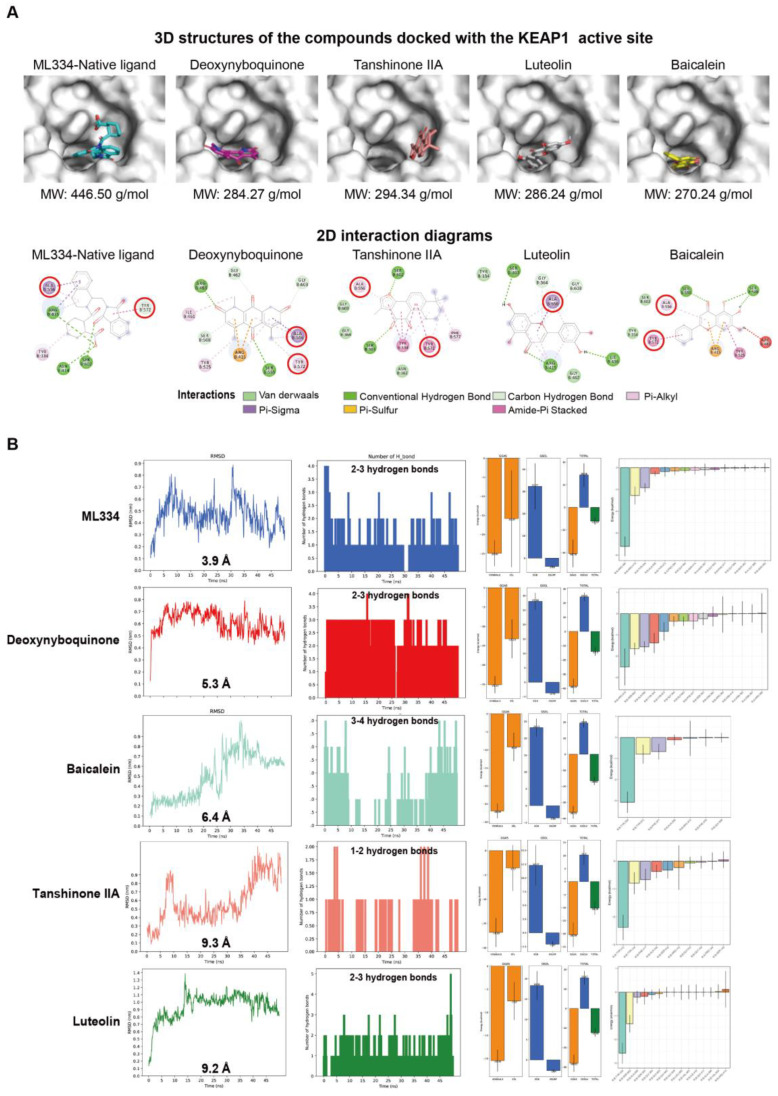
In silico evaluation assessing binding potential of predicted compounds with KEAP1. (**A**) Binding mode and interaction of compounds and activity center of KEAP1. (**B**) Molecular dynamics simulation of positive control and anticipated active compounds. Left to right: RMSD of carbon α of protein compared with initial equilibrium structure; RMSD of ligands to KEAP1 protein; number of hydrogen bonds formed with standard hydrogen bond length and bond angle; MMPB(GB)SA binding energy distribution (ΔGGas (van der Waals and electrostatic energies), ΔGSolid (potential energy of polar solvent and non-polar solvent), and ΔG_Total_ (ΔGGas_Total_ and ΔGSolid_Total_); energy contribution of each residue. Top to bottom: ML334, deoxynyboquinone, baicalein, tanshinone II A, and luteolin.

KEAP1 is considered the key potential therapeutic target in the pathway of the NRF2 regulation of the oxidative stress response [52,53]. Molecular docking was applied to primarily evaluate the potential of the predicted compounds (tanshinone IIA, luteolin, and baicalein) to bind with KEAP1. The result indicates that the compounds tanshinone IIA, luteolin, and baicalein possess favorable docking binding energy with the KEAP1, as presented in Table 2. Detailed docking analysis results are shown in Figure 7A, and the results indicate that tanshinone IIA and baicalein may similarly bind with KEAP1 compared to the native ligand ML334 and deoxynyboquinone by interacting with the residues ALA556 and TYR572. However, only ALA 556 has the same binding site of luteolin with KEAP1 compared to ML334 and deoxynyboquinone, which indicate that the binding mode of luteolin may not be similar to ML334 and deoxynyboquinone.

We further conducted MD simulations to address the flexibility and stability of interactions between anticipated active compounds. The RMSD of carbon α of protein compared with the initial equilibrium structure shows that the binding between ligands and proteins are convergent to equilibrium. In an equilibrium status, accordingly, the average RMSD value between KEAP1 and ML334 in the last 5 ns of simulation is around 3.9 Å, and the distance to deoxynyboquinone is around 5.3 Å. Baicalein shows the nearest distance to KEAP1 at around 6.4 Å compared with tanshinone II A at around 9.3 Å and luteolin at around 9.2 Å. ML334 and deoxynyboquinone are shown to form 2–3 hydrogen bonds with KEAP1. Baicalein is shown to form 3–4 hydrogen bonds with KEAP1 in the pocket, which is the highest number compared to the 1–2 hydrogen bonds formed by tanshinone IIA and the 2–3 bonds formed by luteolin, respectively, and it is even more than the positive control. The MMPB(GB)SA analysis indicates that baicalein exhibits a more stable interaction with KEAP1 compared to ML334 and deoxynyboquinone, with the latter two compounds showing less favorable results. In addition, the most frequent residues whose compounds interact with KEAP1 at the pocket are ALA556, TYR572, TYR334, and ARG415, despite luteolin showing fewer interactions with TYR572 and ARG415, which corresponds to the molecular docking results (Figure 7B and Table 2). These results show that baicalein is most likely to bind with KEAP1 as its direct target.

### 3.5. The Active Compound Baicalein of XBJ Directly Target KEAP1 Protein

**Figure 8:**
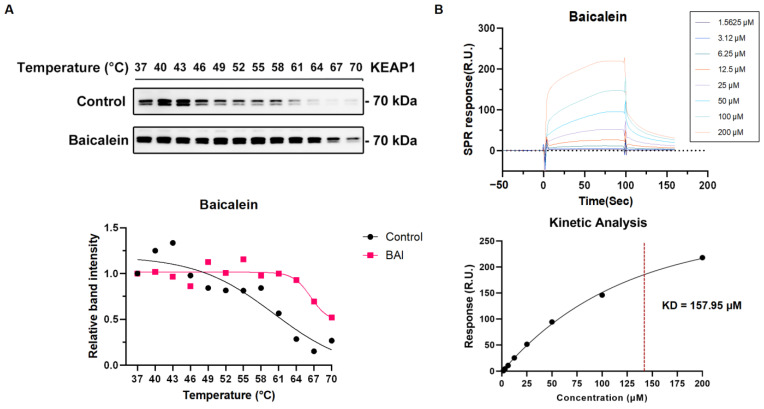
The on-target validation of binding affinity of baicalein with KEAP1. (**A**) The indicated protein specificity was analyzed by Western blotting using KEAP1 antibody. The protein extracted from the MCF-7 cell line lysate was used for CETSA-WB in vitro. (**B**) An SPR analysis of baicalein and KEAP1 protein.

To verify the molecular docking and MD simulation results, we used CETSA-WB to assess the thermal stability of KEAP1 in the presence of baicalein and tanshinone IIA over a range of temperatures. In the control group (treated with DMSO), KEAP1 displayed decreasing stability as the temperature increased. In contrast, KEAP1 in the baicalein-treated group showed markedly higher stability at elevated temperatures, particularly between 64 °C and 70 °C. This indicates that baicalein enhances the thermal stability of KEAP1, suggesting a strong and stable interaction between baicalein and the KEAP1 protein (Figure 8A). Meanwhile, KEAP1 in the tanshinone IIA-treated group did not show higher stability at elevated temperatures. This indicates that tanshinone IIA did not significantly enhance the thermal stability of KEAP1, suggesting no significant interaction between tanshinone IIA and the KEAP1 protein (Figure S7).

An SPR experiment was then utilized to measure the real-time binding interaction between baicalein, tanshinone IIA, and KEAP1. The SPR data show that baicalein binds to KEAP1 in a concentration-dependent manner, as evidenced by the increasing SPR response with higher concentrations of baicalein (from 1.5625 μM to 200 μM). A dissociation constant (KD) of 157.95 μM was determined from the SPR curves, indicating the affinity between baicalein and KEAP1 (Figure 8B). However, the SPR reaction did not change significantly with an increasing tanshinone IIA concentration (from 1.5625 μM to 50 μM). A dissociation constant (KD) of 164.53 M was determined from the SPR curves, indicating no significant affinity between tanshinone IIA and KEAP1 (Figure S8).

### 3.6. Baicalein Activates Antioxidative Response via KEAP1/NRF2 Pathway

**Figure 9:**
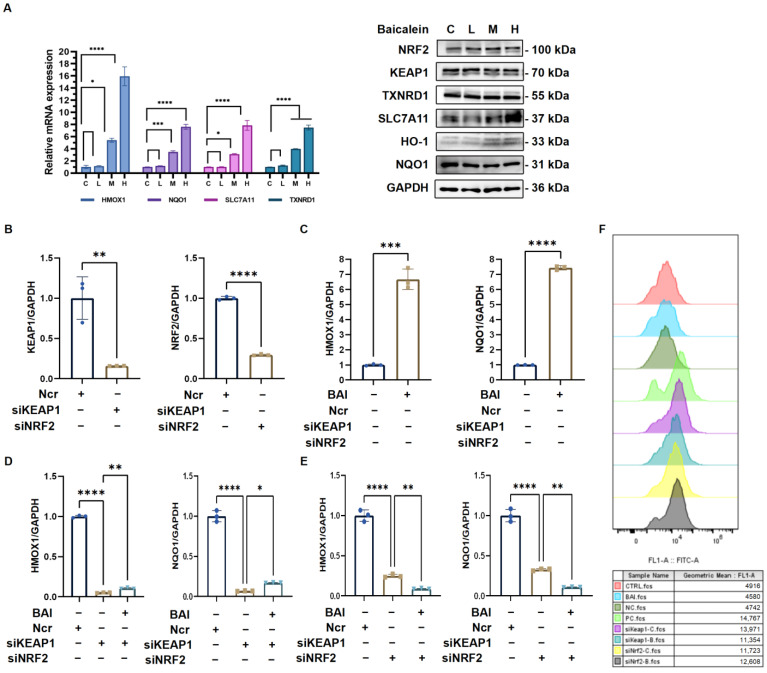
QPCR and Western blot results. (**A**) Gene and protein expression in MCF-7 cell lines treated by baicalein. * *p* < 0.05 vs. CTRL; ** *p* < 0.01 vs. CTRL; *** *p* < 0.001 vs. CTRL; **** *p* < 0.0001 vs. CTRL. MCF-7 cell lines were transfected with ncr, siKEAP1, or siNRF2. Cells were then treated with baicalein (8.772 mg/mL) for 24 h. (**B**) siKEAP1 and siNRF2 knockdown silencing efficiency. (**C**) Control groups with no drug or baicalein. (**D**) siKEAP1 groups with drug and untreated drug. (**E**) siNRF2 groups with drug and untreated drug. * *p* < 0.05; ** *p* < 0.01; *** *p* < 0.001; **** *p* < 0.0001. (**F**) ROS production. Control group (red), baicalein group (blue), NCR group (dark green), positive control (light green), siKEAP1 with no drug (purple), siKEAP1 with baicalein (dark blue), sNRF2 with no drug (yellow), siNRF2 with baicalein (gray).

The mRNA expression in Figure 9A shows the key genes (*HMOX1*, *NQO1*, *SLC7A11*, and *TXNRD1*) involved in the NRF2 pathway across different treatment groups with baicalein at concentrations labeled as control (C), low (L), medium (M), and high (H). The bar graph indicates a significant upregulation of these genes in response to baicalein, especially at higher concentrations, suggesting a dose-dependent activation of the NRF2 pathway. Consistent with the qPCR results, increased protein levels correspond to higher baicalein doses. These findings suggest that baicalein modulated the NRF2-KEAP1 pathway and induced the expression of HO-1 and other antioxidant enzymes.

The silencing efficacy of *KEAP1* and *NRF2* was then verified using knockdown siRNA, as shown in Figure 9B. Baicalein can significantly increase the expression of *HMOX1* and *NQO1*, two important downstream targets of *NRF2* (Figure 9C). We also verified that *HMOX1* and *NQO1* gene expression could be decreased in MCF-7 cell lines by blocking *KEAP1* or *NRF2*. Compared to the NCR group, *HMOX1* and *NQO1* were reduced when *KEAP1* or *NRF2* were silenced. The expression of *HMOX1* and *NQO1* in MCF-7 cell lines with siKEAP1 or siNRF2 knockdown was not reversible by baicalein (Figure 9D,E). Then, ROS production was measured using a flow cytometer. Baicalein can reduce ROS compared with the control group. However, after silencing *KEAP1* and *NRF2*, ROS production was increased (Figure 9F). Together, these results provide a comprehensive overview of how baicalein modulates gene and protein expression related to oxidative stress and antioxidant response through the NRF2 pathway.

## 4. Discussion

Herbal medicines contain a diverse array of chemical constituents. It is widely hypothesized that only specific compounds with drug-like properties contribute significantly to their therapeutic effects. Identifying these active compounds within the herbal mixture is crucial for elucidating the drug’s mechanism of action, optimizing the formulation for enhanced safety and efficacy, and facilitating potential regulatory approval.

In this study, we integrated network pharmacology and RNA sequencing to identify bioactive compounds in XBJ, focusing on their potential to modulate the NRF2/KEAP1/HO-1 pathway, a key regulator in oxidative stress and inflammation. RNA sequencing was first conducted to detect differentially expressed genes (DEGs), revealing transcriptional changes induced by XBJ. We found that genes such as *HMOX1*, *SLC7A11*, *NQO1*, and *TXNRD1* were significantly upregulated, all of which are closely associated with the KEAP1/NRF2 pathway. Furthermore, a bilayer “CS-TPT” network was constructed and analyzed to identify potential active compounds, followed by molecular docking and molecular dynamics to assess their binding affinities. Subsequent CETSA-WB and SPR experiments confirmed that baicalein, one of the identified compounds, exhibited notable binding affinity with KEAP1. This was further validated by silencing KEAP1 and NRF2.

These results emphasize the role of XBJ in mitigating oxidative stress through the NRF2/KEAP1/HO-1 pathway. Given the established importance of HO-1 in oxidative stress and inflammation, especially in viral infections like COVID-19, understanding the molecular mechanisms underlying XBJ’s action is crucial. The following discussion expands on the broader implications of ROS and HO-1 in disease pathology, linking it back to the therapeutic potential of XBJ.

Our findings align with previous research highlighting the role of ROS in disease exacerbation through tissue damage, thrombosis, and red blood cell dysfunction [54]. HO-1, a well-established inducible stress protein, has been shown to mitigate oxidative stress and inflammation by promoting the release of molecules with antioxidant and anti-inflammatory properties [55]. Previous studies have demonstrated that HO-1 upregulation in macrophages leads to reduced pro-inflammatory molecule expression and increased anti-inflammatory cytokines [56]. Notably, in the context of severe COVID-19, a transcriptomic analysis of lung biopsies from patients revealed the suppression of the NRF2 pathway in response to SARS-CoV-2 infection, marked by the reduced expression of HO-1 and NQO1, which are proteins regulated by NRF2 [48]. Moreover, HO-1 induction has been linked to antiviral properties via elevated biliverdin, a byproduct of heme breakdown. Activation of the NRF2/HO-1 pathway has been shown to inhibit viral replication in various cell lines [57].

KEAP1 targets NRF2 for degradation via the ubiquitin–proteasome system, maintaining low NRF2 levels under normal conditions [58]. KEAP1 negatively regulates NRF2 by binding to the Neh2 domain of NRF2, inhibiting its transactivation potential [59]. It is proposed that compounds that activate the antioxidant response disrupt KEAP1/NRF2 binding, leading to NRF2 release and its subsequent nuclear translocation [60]. KEAP1 has also been found to mediate the repression of NRF2 by specifically binding to the amino-terminal Neh2 domain of NRF2 [58]. XBJ, as a multi-target medication, has been shown to upregulate HO-1 at both the mRNA and protein levels through RNA-seq analysis [17,61]. However, further research is needed to identify the direct targets and active compounds that are responsible for this upregulation.

Herein, in order to achieve this, we proposed a new approach utilizing a network analysis to incorporate extensive data and information pertaining to the utilization of traditional Chinese medicines. Notably, we found that baicalein derived from Chishao and Honghua in XBJ could target KEAP1, resulting in the activation of the NRF2/KEAP1 pathway, which leads to the upregulated HO-1. Research has demonstrated that baicalein possesses broad antiviral ability against various viruses [9,62,63]. Moreover, Si-Qin et al. found that baicalein modulates the NRF2/KEAP1 system to exert cytoprotection and cancer chemoprevention [64].

In addition to the active compounds identified in XBJ, naturally occurring bioactive compounds such as flavonoids, polyphenols, chalcones, and statins have shown promising potential in mitigating severe COVID-19 complications, particularly cytokine storm syndrome (CSS). Numerous experimental and preclinical studies have demonstrated their anti-inflammatory, antiviral, and immunomodulatory properties, which can help regulate the hyperactive immune response associated with CSS. For instance, Jo et al. (2020) demonstrated that three flavonoids—herbacetin, rhoifolin, and pectolinarin—inhibit SARS-CoV 3CL protease activity. Their binding mechanisms were confirmed using fluorescence-based methods and an induced-fit docking analysis, providing a basis for developing improved inhibitors against SARS-CoV-2 [65]. Another study by Wan et al. (2021) showed that the synergistic combination of andrographolide and baicalein effectively inhibits SARS-CoV-2 viral entry, protease activity, and the elevation of pro-inflammatory cytokines, as verified through viral pseudovirus assays, protease activity assays, and in vivo inflammation models [66]. This combination was found to be more effective than the individual compounds. Additionally, Liao et al. (2022) introduced a lymphatic-targeting self-microemulsifying drug delivery system containing baicalein (BAPC-SME). This system was shown to effectively inhibit cytokine storm by significantly reducing plasma cytokine levels and alleviating lung tissue damage in mice, as validated through cytokine profiling, histopathological analysis, and distribution studies in vivo [67]. While these studies provide promising preclinical evidence, clinical trials are still needed to confirm the efficacy and safety of these compounds in treating severe COVID-19 cases. The ability of bioactive compounds, like flavonoids, to modulate the immune response could open new therapeutic avenues, especially in mitigating the cytokine storm, but further clinical data are essential to translate these findings into clinical practice.

**Figure 10:**
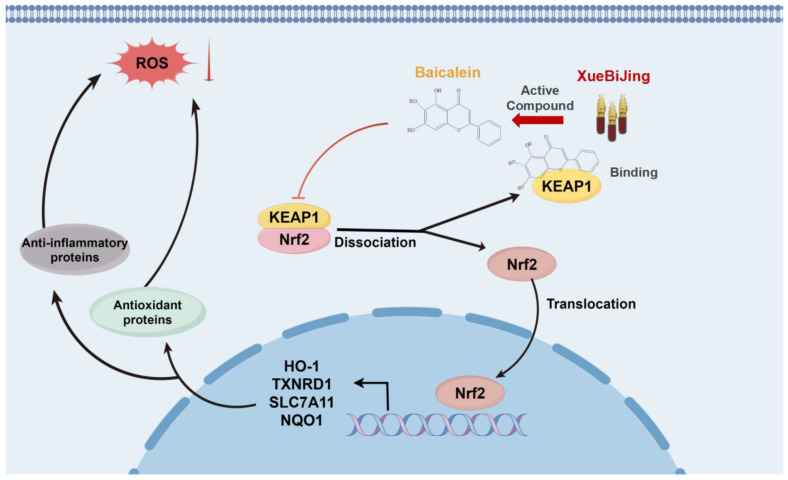
The molecular mechanism of XBJ’s active compound baicalein in the NRF2/KEAP1 pathway.

Our study elucidates the mechanism of baicalein in XBJ, which binds to KEAP1, promoting NRF2 translocation to the nucleus (Figure 10). To identify specific molecular interactions, we utilized molecular docking and molecular dynamics simulations, which revealed strong binding affinities between baicalein and KEAP1 at residues ALA556, TYR572, TYR334, and ARG415. These findings were further validated by CETSA-WB and SPR experiments, confirming baicalein’s capacity to bind KEAP1, thus promoting the activation of the NRF2/KEAP1/HO-1 pathway. These results provide compelling evidence of the molecular basis for XBJ’s therapeutic efficacy. This interaction upregulates the expression of genes like HO-1 and NQO1, which are involved in immune modulation and antioxidant defense. This mechanism could have therapeutic implications for treating infectious diseases. Future studies will investigate baicalein’s therapeutic effects on diseases such as breast cancer and sepsis, with a focus on the NRF2/KEAP1 pathway [68,69].

One limitation of this study is the reliance on MCF-7 cells to validate our hypothesis. While MCF-7 cells are widely used as a cell model for drug screening, including extensive applications in resources such as ConnectivityMap (CMap), which aligns with the methodological framework of our study, they may not fully replicate the complex systemic conditions found in human patients. To address this limitation, future research should further investigate the effects of XBJ’s active compounds in immune cells and clinical samples. Such studies would provide a more comprehensive understanding of the therapeutic potential of XBJ and its bioactive components. We believe that this approach will be crucial for translating our findings into clinically relevant applications.

## 5. Conclusions

This study shows that baicalein binding to KEAP1 activates the NRF2/KEAP1 signaling pathways linked to antioxidative stress and anti-inflammatory biological processes. Our research on the beneficial effects of XBJ uncovers its mechanisms and provides a helpful framework for analyzing the intricate active ingredients in TCMs. Furthermore, the integrated methods in this study provide a new dimension for studying virus–immune system dynamics via a pathway analysis of drug molecules.

## Figures and Tables

**Table 1:** Primers used for qPCR validation.

	Forward Primer (5′–3′)	Reverse Primer (5′–3′)
*GAPDH*	ACCCACTCCTCCACCTTTGAC	TGTTGCTGTAGCCAAATTCGTT
*HMOX1*	AAGACTGCGTTCCTGCTCAAC	AAAGCCCTACAGCAACTGTCG
*SLC7A11*	TCTCCAAAGGAGGTTACCTGC	AGACTCCCCTCAGTAAAGTGAC
*NQO1*	GAAGAGCACTGATCGTACTGGC	GGATACTGAAAGTTCGCAGGG
*TXNRD1*	ATATGGCAAGAAGGTGATGGTCC	GGGCTTGTCCTAACAAAGCTG
*NRF2*	TCAGCGACGGAAAGAGTATGA	CCACTGGTTTCTGACTGGATGT
*KEAP1*	CTGGAGGATCATACCAAGCAGG	GGATACCCTCAATGGACACCAC

**Table 2:** The docking and simulation results of the predicted active compounds with KEAP1.

Target Protein	Compounds	PubChem CID	Molecular Weight (g/mol)	Structure	Source	Binding Energy	Ligand–Protein RMSD (Å)	MMPB (GB)SA ΔG_Total_ (kcal/mol)	Number of Hydrogen Bonds
KEAP1 (PDB ID: 4L7B)	ML 334	56840728	446.5	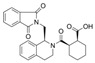	Native ligand	−10.8	3.9	−12.19	2–3
	Deoxynyboquinone	295934	284.27	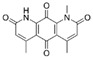	NRF2 activator	−8.7	5.3	−14.1	2–3
	Tanshinone IIA	164676	294.3	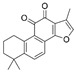	Danshen	−8.6	9.3	−10.19	1–2
	Luteolin	5280445	286.24	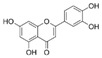	Honghua	−8.6	9.2	−12.35	2–3
	Baicalein	5281605	270.24	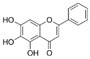	Honghua/Chishao	−8.2	6.4	−16.65	3–4

## Data Availability

Data is contained within the article and Supplementary Material.

## Supplementary Materials

The following supporting information can be downloaded at: https://www.mdpi.com/article/10.3390/antiox14030248/s1, Figure S1. The matrix ***A^m×n^*** of compound (***ci***) and target (***bj***). Figure S2. The matrix ***E^p×q^*** of the compound module (*m_u_*) and the target module ($m_{v}^{*}$). Figure S3. A diagram for illustrating the bilayer network analysis of XBJ for predicting the potential active compounds. Figure S4. Compounds characterization of XBJ. Figure S5. Secondary mass spectrum of baicalein. Figure S6. MCF-7 cells were transfected with siKEAP1 for 48 h, mRNA level was assayed by qRT-PCR. Figure S7. CETSA-WB assessed the thermal stability of KEAP1 in the presence of tanshinone IIA over a range of temperatures. Figure S8. SPR experiment was utilized to measure the real-time binding interaction between tanshinone IIA and KEAP1. Table S1 Experimental Results of zebrafish. Table S2 siRNA oligo list. Table S3 Identification results of XBJ by UPLC-Q-TOF-MS/MS. Table S4 Enrichment analysis report. Table S5 Calculated compound’s contribution ***C(i)uv*** to the selected target TPT_module_1. References [70,71,72,73,74,75,76] are cited in the supplementary materials.

## Abbreviations

The following abbreviations are used in this manuscript:

XBJ	XueBiJing injection
ROS	Reactive oxygen species
KEAP1	Kelch-like ECH associating protein 1
NRF2	Nuclear factor erythroid 2-related factor 2
HO-1	Heme-induced heme oxygenase-1
DEGs	Differentially expressed genes
MPX	Myeloperoxidase
CS network	Compound similarity network
TPT network	Target–pathway–target network
MD	Molecular dynamics
RMSD	Root mean square deviation
SPR	Surface plasmon resonance
WB	Western blotting
CETSA	Cellular thermal shift assay
NCR	Non-coding RNA
siNRF2	siRNA against Nrf2
siKEAP1	siRNA against KEAP1
BAI	Baicalein
