# Supplementary material for: Identifying Baicalein as a Key Bioactive Compound in XueBiJing Targeting KEAP1: Implications for Antioxidant Effects
Source: Antioxidants (Basel). 2025 Feb 20;14(3):248. doi: 10.3390/antiox14030248 (PMC11939276; doi:10.3390/antiox14030248)
Supplement: Supplementary file 1 [file antioxidants-14-00248-s001.zip › Supplementary information_clean.pdf]

Supplementary information

Identification of Baicalein As a Key Bioactive Compound in XueBiJing Targeting KEAP1: Implications for Antioxidant Effects

Ting-Syuan Lin [1,2,3,†], Xiao-Xuan Cai [1,2,3,†], Yi-Bing Wang [1,2,3,†], Jia-Tong Xu [1,2,3], Ji-Han Xiao [1], Hsi-Yuan Huang [1,2,3], Shang-Fu Li [1,2,3], Kun-Meng Liu [4], Ji-Hang Chen [1], Li-Ping Li [1,2,3], Jie Ni [1,2,3], Yi-Gang Chen [1,2,3], Zi-Hao Zhu [1,2,3], Jing Li [1,2,3], Yuan-Jia Hu [5,6], Hsien-Da Huang [1,2,3,7], Yang-Chi-Dung Lin [1,2,3,*], Hua-Li Zuo [1,2,3,*]

[1] School of Medicine, The Chinese University of Hong Kong, Shenzhen, Guangdong 518172, P.R. China
[2] Warshel Institute for Computational Biology, School of Medicine, The Chinese University of Hong Kong, Shenzhen, Guangdong 518172, P.R. China
[3] Guangdong Provincial Key Laboratory of Digital Biology and Drug Development, The Chinese University of Hong Kong, Shenzhen, Guangdong, P.R. China
[4] Center for Medical Artificial Intelligence, Shandong University of Traditional Chinese Medicine, Qingdao 266112, China
[5] State Key Laboratory of Quality Research in Chinese Medicine, Institute of Chinese Medical Sciences, University of Macau, Macao 999078, China
[6] Department of Public Health and Medicinal Administration, Faculty of Health Sciences, University of Macau, Macao 999078, China
[7] Department of Endocrinology, Key Laboratory of Endocrinology of National Health Commission, Peking Union Medical College Hospital, Chinese Academy of Medical Sciences & Peking Union Medical College, Beijing, 100730, P.R. China
***** Correspondence: zuohuali@cuhk.edu.cn (H.L.Z.); yangchidung@cuhk.edu.cn (Y.-C.-D.L.)
Tel.: +86-0755-23518211 (H.L.Z.); +86-0755-84273216 (Y.-C.-D.L.)
[†] These authors contributed equally

Figure S1. The matrix $\boldsymbol{A^{m\times n}}$ of compound (***ci***) and target (***bj***).

Figure S2. The matrix $\boldsymbol{E^{p\times q}}$ of the compound module (m_u) and the target module (m_v^*).

Figure S3. A diagram for illustrating the bilayer network analysis of XBJ for predicting the potential active compounds.

Figure S4. Compounds characterization of XBJ.

Figure S5. Secondary mass spectrum of baicalein.

Figure S6. MCF-7 cells were transfected with siKEAP1 for 48h, mRNA level was assayed by qRT-PCR.

Figure S7. CETSA-WB assessed the thermal stability of KEAP1 in the presence of tanshinone IIA over a range of temperatures.

Figure S8. SPR experiment was utilized to measure the real-time binding interaction between tanshinone IIA and KEAP1.

Table S1 Experimental results of zebrafish.

Table S2 siRNA oligo list.

Table S3 Identification results of XBJ by UPLC-Q-TOF-MS/MS.

Table S4 Enrichment analysis report.

Table S5 Calculated compound's contribution ***C(i)uv*** to the selected target TPT_module_1.

1. Materials and Methods

1.1. LC-MS/MS analysis of XBJ

1.1.1. Instruments

(1) Ultra-High Performance Liquid Chromatography (UHPLC): Shimadzu Nexera UHPLC LC-30A.
(2) Column: Shim-pack GIST 2 μm C18, 2.1 × 100mm, P/N: 227-30001-04; S/N: 21E 24484.
(3) Mass Spectrometry: TripleTOF 6600+, Sciex.
(4) Vacuum Concentrator: Auto R3, Jiaimu Technology Co., Ltd., Beijing, China.

1.1.2. Reagents

(1) XBJ samples: The XBJ manufactured by Tianjin Chase Sun Pharmaceutical Co., Ltd., and was purchased from Shijiazhuang Kang Ren Tang Pharmaceutical Co., Ltd, China (No.2107191).
(2) Standard compounds: Oxypaeoniflorin (No. DY0074-0020, molecular weight (MW): 496.46 g/mol), senkyunolide I (No. DY0009-0020, MW: 224.25 g/mol), luteolin (No. DM0032-0020, MW: 286.24 g/mol), tanshinone IIA (No. DD0011, MW: 294.34 g/mol) and baicalein (No. DH0024-0020, MW: 270.24 g/mol), with a purity of 98%, were purchased from Chengdu Desite Biological Technology Co. Ltd, China.
(3) Acetonitrile was purchased from Fisher Chemical (A998-4; LOT: F22M1C201).
(4) Ammonium Hydroxide was obtained from Fisher Scientific (No. A669-500).
(5) Ultra-pure water was prepared using the Direct-Q3 Water Purification System (Merck Millipore).

1.1.3. Chromatographic Conditions

Samples were analyzed using an UHPLC system coupled with an Sciex TripleTOF 6600+ mass spectrometer. Separation was performed on a C18 column (2 μm C18, 2.1 × 100mm). Mobile phase A was 99.9% water, and 0.01% ammonium hydroxide, while mobile phase B was 100% acetonitrile. Gradient elution was conducted with the following program: 0-2 min, from 0% to 5% B; 2-22 min, 99% B; 22-26 min, 99% B; 26-30 min, from 99% to 5% B. Flow rate: 0.3 mL/min; column temperature: 30°C; injection volume: 20 μL.

1.1.4. Mass Spectrometry Conditions

(1) Positive mode

Mass spectrometry analysis was performed using an Sciex TripleTOF 6600 Plus system with the following parameters: curtain gas (CUR) set to 35 psi, gas source 1 (GS1) at 55 psi, gas source 2 (GS2) at 55 psi, ion spray voltage (ISVF) at 5500 V, and ion source temperature (TEM) at 550°C.

(2) Negative mode

Mass spectrometry analysis was performed using an Sciex TripleTOF 6600 Plus system with the following parameters: curtain gas (CUR) set to 35 psi, gas source 1 (GS1) at 55 psi, gas source 2 (GS2) at 55 psi, ion spray voltage (ISVF) at -4500 V, and ion source temperature (TEM) at 550°C.

1.1.5. Preparation of reference compounds

Accurately weigh each reference standard and prepare a stock solution at a concentration of 1 mg/mL using methanol. Aliquots of each stock solution were precisely measured and combined to prepare a mixed standard solution using the initial mobile phase. The mixed solution was then diluted to concentration of 2.5 ug/mL.

1.1.6. Preparation of XBJ

Five milliliters of XBJ were concentrated to dryness using a vacuum centrifuge and reconstituted in 500 μL methanol, resulting in a 10-fold concentration.

1.2. Network Analysis

1.2.1. The compound and target profile of XBJ

Chemical databases provided the compounds used by XBJ, and literature mining was used to augment the information. Pharmacology from traditional Chinese medical systems is included in the chemical databases[1] (TCMSP, http://lsp.nwu.edu.cn/tcmspsearch.php), and the HERB[2] (http://herb.ac.cn/). Common amino acids and high molecular weight substances like polysaccharides were excluded from the study. The compounds' names and Chemical Abstracts Service (CAS) numbers were matched after the compounds were standardized using SciFinder (https://scifinder.cas.org). Additionally, using their chemical names or structures, the PubChem CIDs and canonical SMILES string of these compounds were obtained from PubChem (https://pubchem.ncbi.nlm.nih.gov/). The canonical SMILES string was used to weed out duplicate entries. Compounds lacking any relevant targets that meet our criteria as introduced below were not included in this study in order to map the target profile in the network-based analysis.

We used three prediction tools, including SuperPred[3] (https://prediction.charite.de/index.php), SEA[4] (http://sea.bkslab.org/) and TCMSP[1] (https://tcmsp-e.com/tcmsp.php), to find their potential targets. We applied different filters to each tool's results: for SuperPred, we only kept predicted targets with score > 0.8 and known targets; for SEA, we only kept targets with p<e-12 or Tc>0.59; for TCMSP, we kept all targets. We then combined the results and gave each target a final score based on the confidence of the methods. A target got one point for each prediction by SEA or SuperPred, and two points for each prediction by TCMSP or inclusion in SuperPred known targets. Uniformity was achieved for all targets through the use of UniProt ID and gene name. Then, in order to gather important target-pathway interactions required for the ensuing network building and analysis, an enrichment analysis was carried out. Using MetaCore™ (©2022 Clarivate Analytics), the targets were enriched to pathways to create the target–pathway interactions. For additional study, we chose pathways with enriched p-value < 0.05.

1.2.2. Compound similarity (CS) network

Compound similarity searches based on fingerprints were conducted using RDkit. The CS network, in which the nodes represent compounds and the edges represent associations, was established by extracting the compounds with a Tanimoto coefficient (Tc) ≥ 0.8. Compounds are grouped according to their structural similarity in CS network. In a CS network, compounds are grouped based on how similar their structures are. Among every pair of substances having a Tc value of at least 0.8. This indicates that compounds connected by edges exhibit significant structural similarities. With a resolution of 1.0, the Louvain algorithm built into Gephi was utilized to identify and split the clusters within the CS network. We performed independent analyses on the structures of nodes (compounds) within each cluster using the chemical data from PubChem[5].

1.2.3. Target-pathway-target (TPT) network

The target-(pathway)-target network, or TPT network, was created as a result of the integration of target-pathway interactions[5]. Using Pajek software, the one-mode target-target interactions that result from the two-mode target-pathway relationship will serve as the foundation for the initial construction of the TPT network. The target-pathway interactions will then be translated into target-based associations based on pathways. Topologically speaking, these relationships can be thought of as a network. By letting N be a set of nodes that represent targets and E be a set of edges, where elements of E are unordered pairs of distinct nodes n_i, n_j representing a pathway-based link between a target pair, one can mathematically obtain a familiar representation[6]. The two sets together are called a simple network $G = (N, E)$. The transformation of the two-mode target–pathway interactions into one-mode target–target interactions were accomplished through the utilization of Pajek software. Subsequently, the resulting target–target interactions were visually represented using Gephi software[7], thereby generating the TPT network of XBJ. Within this network, the nodes represent protein targets, and the presence of an edge connecting any pair of nodes signifies their involvement in at least one shared pathway.

1.2.4. Network analysis of bilayer CS−TPT network

In the bilayer network of CS−TPT, there is a chemical layer composed of a CS network, followed by a biological layer composed of a TPT network. The interactions between compounds and targets determine how those two layers are connected.

Network modules, also called groups, clusters, or communities, are widely used to investigate the underlying relationships between herbal remedies, chemical clusters with comparable structures or bioactivities, or proteins with comparable roles. Based on extant literature and our own earlier studies, it has been noted that biological and chemical networks related to TCMs display clustering properties. This suggests that each network's nodes are easily arranged into groups of nodes, with each group exhibiting dense internal connectivity. This implies that network modules consist of nodes that are highly interconnected. The identification of these modules is of great importance as it has the potential to uncover hidden structural information within the network. To investigate the modularity structure of this project, the Louvain algorithm module detection, integrated into Gephi, was employed. Furthermore, the following illustrated network-based method is an update of our previous work.

Matrix or graph representations are two ways to show networks. The study will employ the following types of matrices in order to make the algorithms more understandable. This section will detail the process of calculating the weighted relationships between the targets and sample compounds (Figure S1.).

A	b_1	$\cdots$	b_j	$\cdots$	b_n
c_1	a_{11}	$\cdots$	a_{1j}	$\cdots$	a_{1n}
$\vdots$	$\vdots$	$\vdots$	$\vdots$	$\vdots$	$\vdots$
c_i	a_{i1}	$\cdots$	$\boldsymbol{a_{ij}}$	$\cdots$	a_{in}
$\vdots$	$\vdots$	$\vdots$	$\vdots$	$\vdots$	$\vdots$
c_m	a_{m1}	$\cdots$	a_{mj}	$\cdots$	a_{mn}

Figure S1. The matrix $\boldsymbol{A^{m \times n}}$ of compound (***ci***) and target (***bj***).

Where a_{ij} = 1 indicates a relationship between the compound (c_i) and the target (b_j), while 0 indicates no relationship. The work in section 1 is where the a_{ij} data originates. Drawing from existing literature and our prior research, it is evident that an herbal network typically exhibits a non-random, scale-free structure, displaying small world properties. Consequently, it is imperative to acknowledge the distinctiveness of compounds within a chemical network and targets within a biological network. To quantify the strength of the connection between compound c_i and target b_j, Equation (1) provides a means to compute the weighted value d_{ij}.

$$d_{ij} = w(c_i) \cdot a_{ij} \times w(b_j) \cdot a_{ij} \qquad \text{Equation (1)}$$

Where the weight of the target (b_j) in the biological network is indicated by $w(b_j)$, and the weight of the compound (c_i) in the chemical network is indicated by $w(c_i)$. The study examined four widely used indicators of nodal weights: degree centrality, betweenness centrality, closeness centrality, and eigenvector.

Furthermore, it is crucial to take into account the chemical and biological clustering characteristics in order to accurately represent their inherent associations. To do this, the biological and chemical networks must be combined into cluster-based networks, where chemical compounds with similar structures and biological molecules with similar functions are combined, respectively. Thus, as shown in Figure S2 below, the matrix $A^{m \times n}$ can be changed into the matrix $E^{p \times q}$.

E	m_1^*	$\cdots$	m_v^*	$\cdots$	m_q^*
m_1	e_{11}	$\cdots$	e_{1v}	$\cdots$	e_{1q}
$\vdots$	$\vdots$	$\vdots$	$\vdots$	$\vdots$	$\vdots$
m_u	e_{u1}	$\cdots$	e_{uv}	$\cdots$	e_{uq}
$\vdots$	$\vdots$	$\vdots$	$\vdots$	$\vdots$	$\vdots$
m_p	e_{p1}	$\cdots$	e_{pv}	$\cdots$	e_{pq}

Figure S2. The matrix $E^{p \times q}$ of the compound module (m_u) and the target module (m_v^*).

Where e_{uv} is employed to calculate the correlation between the target module in TPT network (m_v^*) and the compound module (m_u) in CS network. In a similar manner, e_{uv} can be determined by adding up d_{ij}.

$$e_{uv} = \sum d_{ij} = \sum_{c_i \in m_u, b_j \in m_v^*} w(c_i) \cdot a_{ij} \times w(b_j) \cdot a_{ij} \qquad \text{Equation (2)}$$

Given that the multi-compounds may act with multi-targets, it follows that multi-compound modules may act with multi-target modules. The strength of the association between a compound module and a target module is reflected by the e_{uv}. Furthermore, for each pair of investigated compound module (m_u)-target module (m_v^*), the every single compound's contribution $C(i)_{uv}$ to the target module can be

evaluated by the following Equation (3)

$$C(i)_{uv} = \frac{e(i)_{uv}}{n(i)_{uv}} \qquad \text{Equation (3)}$$

Where ***e(i)**$_{uv}$* denotes summing strength of the association between the compound i in the compound module ($\boldsymbol{m_u}$) and the target module (m_v^*), the ***n(i)**$_{uv}$* denotes the number of compound-target associations that involved in the investigated compound module ($\boldsymbol{m_u}$)-target module (m_v^*).

Except for the ***C(i)**$_{uv}$* is taken into account in predicting potential active compounds. Furthermore, to limit the range of active compounds for subsequent verification, the potential influenced pathway that the XBJ may regulate, as inferred from the RNA-seq analysis, is also considered.

A brief diagram for illustrating the bilayer network analysis of XBJ for predicting the potential active compounds was shown in Figure S3.

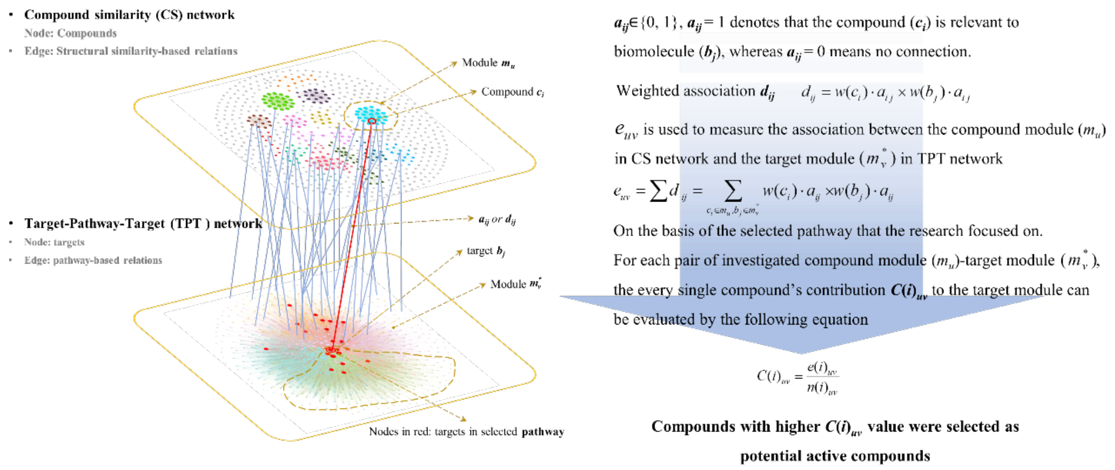

Figure S3. A diagram for illustrating the bilayer network analysis of XBJ for predicting the potential active compounds.

1.3. siRNA oligo design

Table S2 siRNA oligo list.

Name	Type	Sequence
human-NRF2-1	Sense	GGAUUAUUAUGACUGUUAAAU
	Anti-sense	UUAACAGUCAUAAUAAUCCUU
human-NRF2-2	Sense	GAGUUACAGUGUCUUAAUAUU
	Anti-sense	UAUUAAGACACUGUAACUCAG
human-NRF2-3	Sense	CAGUCUUCAUUGCUACUAAUC
	Anti-sense	UUAGUAGCAAUGAAGACUGGG
human-KEAP1-1	Sense	GAGUGUUACGACCCAGAUACA
	Anti-sense	UAUCUGGGUCGUAACACUCCA
human-KEAP1-2	Sense	GGUCAAGUACGACUGCGAACA
	Anti-sense	UUCGCAGUCGUACUUGACCCA
human-KEAP1-3	Sense	GCAAGGACUACCUGGUCAAGA
	Anti-sense	UUGACCAGGUAGUCCUUGCAG
human GAPDH Positive Control (PC)	Sense	GUAUGACAACAGCCUCAAGTT
	Anti-sense	CUUGAGGCUGUUGUCAUACTT
Negative Control (NC)	Sense	UUCUCCGAACGUGUCACGUTT
	Anti-sense	ACGUGACACGUUCGGAGAATT

2. Results

2.1. Compounds characterization of XBJ

As shown in Figure S4, compounds were characterized by LC-MS/MS in XBJ and mixture standard samples. The detail information of 5 compounds was listed in Table S2. The secondary mass spectra of baicalein in XBJ and the standard mixture are shown in Figure S5.

Table S3 Identification results of XBJ by UPLC-Q-TOF-MS/MS.

ID	Rt(min)	Name	Adduct type	Formula	MS (m/z)	MS/MS spectrum
1	5.17	Oxypaeoniflorin	[M-H]-	$C_{23}H_{28}O_{12}$	495.1525	137.0252, 165.0560, 465.1418, 495.1576
2	6.916	Baicalein	[M-H]-	$C_{15}H_{10}O_5$	269.0461	79.9575, 117.0341, 151.0022, 225.0940, 269.0461
3	7.058	Luteolin	[M-H]-	$C_{15}H_{10}O_6$	285.0397	117.0354, 143.0508, 199.0756, 267.1020, 285.0397
4	10.388	Senkyunolide I	[M-H]-	$C_{12}H_{16}O_4$	223.0963	93.0334, 117.0342, 161.0242, 162.0324, 203.0710, 205.0879
5	11.442	Tanshinone IIA	[M+H]+	$C_{19}H_{19}O_3$	295.1541	179.1064, 191.1078, 205.1215, 235.1309, 265.1443, 277.1383, 295.1541

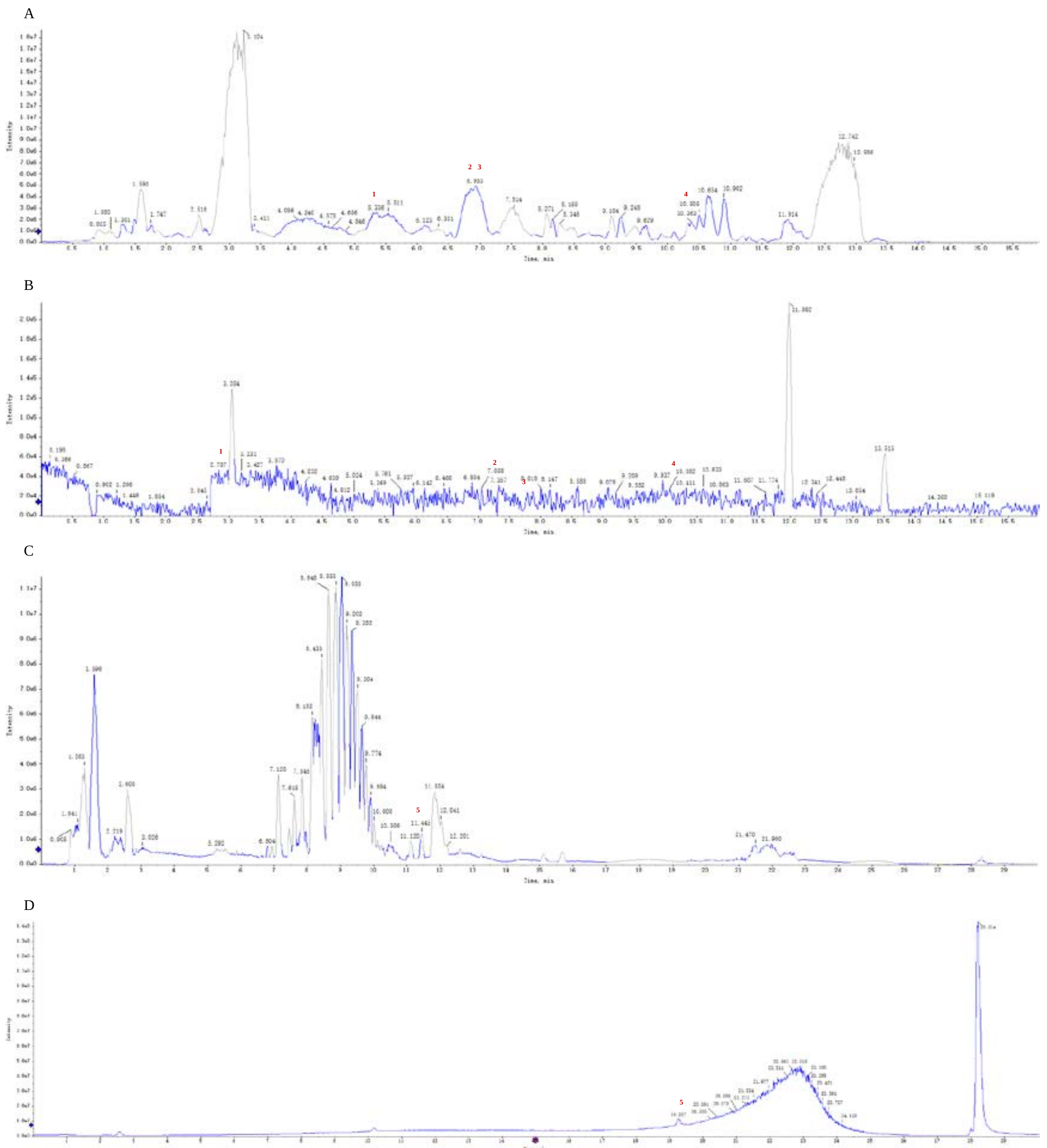

Figure S4. Compounds characterization of XBJ. (A) LC-MS/MS result of the XBJ sample negative mode. (B) LC-MS/MS result of mixed standard samples negative mode. (C) LC-MS/MS result of the XBJ sample positive mode. (B) LC-MS/MS result of mixed standard samples postive mode. 1. Oxypaeoniflorin; 2. Baicalein; 3. Luteolin; 4. Senkyunolide I; 5. Tanshinone IIA.

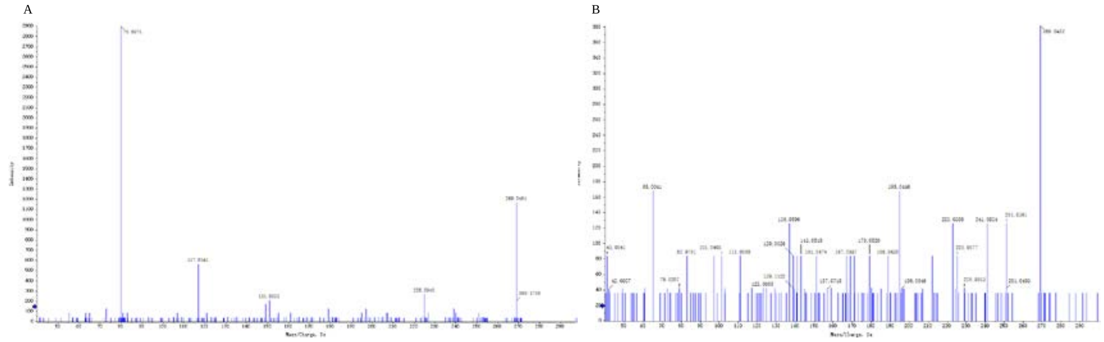

Figure S5. Secondary mass spectrum of baicalein. (A) Secondary mass spectrum of baicalin in XBJ. (B) Secondary mass spectrum of baicalein in standard mixture.

2.2. Network Analysis

Table S5. Calculated compound's contribution ***C(i)uv*** to the selected target TPT_module_1.

Pubchem CID	Compound	CS_Modularity	Class	Gene	Uniprot ID	Property	TPT_Modularity	euv	e(i)uv	C(i)uv
164676	Tanshinone IIA	CS_Module_7	Terpenoids	TP53	P04637	Immune	TPT_Module_1	44.998	4.406	0.14
5280445	Luteolin	CS_Module_12	Flavonoids	GSK3B	P49841	Immune/Coagulation	TPT_Module_1	21.899	4.274	0.097
5280445	Luteolin	CS_Module_12	Flavonoids	TP53	P04637	Immune	TPT_Module_1	21.899	4.274	0.097
5281605	Baicalein	CS_Module_12	Flavonoids	TP53	P04637	Immune	TPT_Module_1	21.899	1.726	0.091
5280443	Apigenin	CS_Module_12	Flavonoids	GSK3B	P49841	Immune/Coagulation	TPT_Module_1	21.899	3.264	0.084
5280443	Apigenin	CS_Module_12	Flavonoids	TP53	P04637	Immune	TPT_Module_1	21.899	3.264	0.084
5280343	Quercetin	CS_Module_12	Flavonoids	GSK3B	P49841	Immune/Coagulation	TPT_Module_1	21.899	5.109	0.076
5280343	Quercetin	CS_Module_12	Flavonoids	TP53	P04637	Immune	TPT_Module_1	21.899	5.109	0.076
5281672	Myricetin	CS_Module_12	Flavonoids	GSK3B	P49841	Immune/Coagulation	TPT_Module_1	21.899	1.586	0.061

2.3. siRNA oligo efficiency

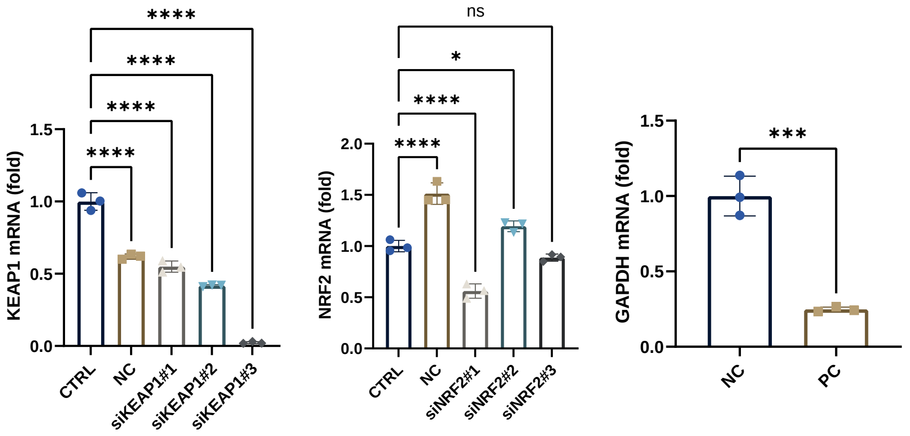

Figure S6. MCF-7 cells were transfected with siKEAP1 for 48h, mRNA level was assayed by qRT-PCR. **p ≤ 0.01, ***p ≤0.001, n.s. = not significant.

2.4. The active compound tanshinone IIA of XBJ directly target KEAP1 protein

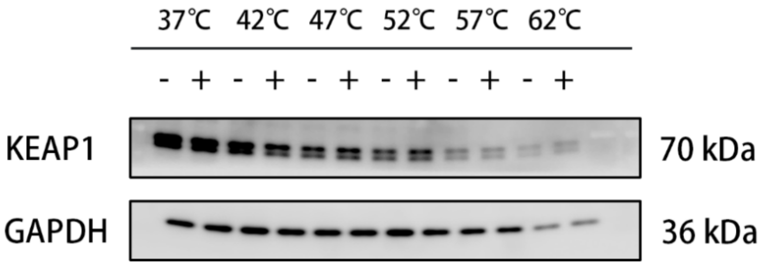

Figure S7. CETSA-WB assessed the thermal stability of KEAP1 in the presence of tanshinone IIA over a range of temperatures.

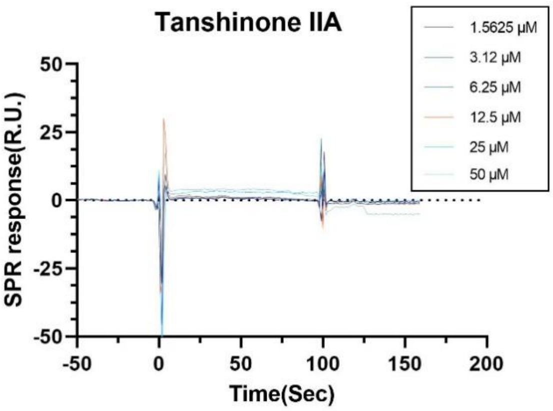

Figure S8. SPR experiment was utilized to measure the real-time binding interaction between tanshinone IIA and KEAP1.

References

1. Ru, J.; Li, P.; Wang, J.; Zhou, W.; Li, B.; Huang, C.; Li, P.; Guo, Z.; Tao, W.; Yang, Y.; et al. TCMSP: a database of systems pharmacology for drug discovery from herbal medicines. *J Cheminform* **2014**, *6*, 13, doi:10.1186/1758-2946-6-13.
2. Fang, S.; Dong, L.; Liu, L.; Guo, J.; Zhao, L.; Zhang, J.; Bu, D.; Liu, X.; Huo, P.; Cao, W.; et al. HERB: a high-throughput experiment- and reference-guided database of traditional Chinese medicine. *Nucleic Acids Res* **2021**, *49*, D1197-D1206, doi:10.1093/nar/gkaa1063.
3. Nickel, J.; Gohlke, B.O.; Erehman, J.; Banerjee, P.; Rong, W.W.; Goede, A.; Dunkel, M.; Preissner, R. SuperPred: update on drug classification and target prediction. *Nucleic Acids Res* **2014**, *42*, W26-31, doi:10.1093/nar/gku477.
4. Keiser, M.J.; Roth, B.L.; Armbruster, B.N.; Ernsberger, P.; Irwin, J.J.; Shoichet, B.K. Relating protein pharmacology by ligand chemistry. *Nat Biotechnol* **2007**, *25*, 197-206, doi:10.1038/nbt1284.
5. Zuo, H.L. An analytical method of pathway-based target networks and its application in modern research of herbal formulae. University of Macau, Macau, 2020.
6. Hassan, M.; Elzallat, M.; Aboushousha, T.; Elhusseny, Y.; El-Ahwany, E. MicroRNA-122 mimic/microRNA-221 inhibitor combination as a novel therapeutic tool against hepatocellular carcinoma. *Noncoding RNA Res* **2023**, *8*, 126-134, doi:10.1016/j.ncrna.2022.11.005.
7. Subelj, L.; Bajec, M. Unfolding communities in large complex networks: combining defensive and offensive label propagation for core extraction. *Phys Rev E Stat Nonlin Soft Matter Phys* **2011**, *83*, 036103, doi:10.1103/PhysRevE.83.036103.
